# Pliocene Forest Fragmentation Shaped Speciation in Tropical Asia's Giant Squirrels (*Ratufa*)

**DOI:** 10.1111/mec.70179

**Published:** 2025-11-25

**Authors:** Arlo Hinckley, Gonzalo E. Pinilla‐Buitrago, Jesús E. Maldonado, Mary Faith C. Flores, Jacob A. Esselstyn, Nurul Inayah, Melissa T. R. Hawkins

**Affiliations:** ^1^ Division of Mammals, Department of Vertebrate Zoology, National Museum of Natural History Smithsonian Institution Washington DC USA; ^2^ Departamento de Zoología Universidad de Sevilla Seville Spain; ^3^ Instituto de Ecología (Unidad Mérida) Universidad Nacional Autónoma de México Mexico City México; ^4^ Center for Conservation Genomics Smithsonian National Zoo and Conservation Biology Institute Washington DC USA; ^5^ Museum of Natural Science and Department of Biological Sciences Louisiana State University Baton Rouge Louisiana USA; ^6^ Museum Zoologicum Bogoriense, Research Center for Biosystematics and Evolution National Research and Innovation Agency Cibinong Indonesia

**Keywords:** genomics/proteomics, mammals, niche modelling, phylogeography, speciation, systematics

## Abstract

Tropical Asia's complex, dynamic geological and climatic history, coupled with its diverse topography, provides a fascinating setting to study evolutionary processes driving high biodiversity. This phylogenomic research reconstructs the evolutionary history of the strictly arboreal and forest‐dependent Oriental Giant Squirrels (*Ratufa*) to gain insights into the interplay between paleo‐forest distribution and regional diversification. By analysing genomic data (complete mitochondrial genomes and approximately 4000 nuclear ultraconserved elements) from historic museum specimens and conducting divergence time estimation and niche modelling, we uncover how global paleoclimate cooling, the uplift of the Himalayas and Tibetan Plateau, and habitat fragmentation led to allopatric speciation in refugia during the mid‐Miocene, Miocene–Pliocene boundary, and late Pliocene, in synchrony with other evergreen forest‐dependent species. Our findings underscore the potential role of grassland expansion during climatic oscillations and the North Sunda and Mekong paleorivers in isolating populations and promoting vicariance and speciation in this region. This research suggests a species‐level diversity underestimation within *R. bicolor* and *R. affinis* , supporting the recognition of *R. gigantea* as a distinct species, along with several candidate species that warrant integrative taxonomic revision. Additionally, this study highlights the rapid and independent evolution of dwarfism in three *Ratufa* lineages and discusses challenges in museum genomics. Ultimately, this study serves as a valuable reference on the historical biogeography of tropical Asia, providing important insights for the conservation of these threatened taxa and the evolutionary processes that generate and maintain biodiversity in this hyperdiverse region.

## 1 Introduction

Southeast Asia's (SEA) dynamic geological and climatic history, coupled with its complex topography offers a fascinating setting to investigate evolutionary processes that contribute to high levels of biodiversity. SEA biodiversity is believed to have arisen from several diversification processes: (a) adaptation to diverse climates with varying rainfall, temperature, and seasonality; (b) repeated, intermittent dry‐land connections between the continent and Sundaic islands during the Plio‐Pleistocene; (c) the distinct ecological conditions in lowland versus highland habitats, leading to local adaptation and divergence, or acting as refuges/barriers during climate changes; (d) changes in the paths and flows of major rivers altered barriers and corridors, isolating or connecting different lineages (Heaney 1991; Hall 2013; Geissler et al. 2015; Sheldon et al. 2015; Morley 2018; Mason et al. 2019; Cros et al. 2020; Husson et al. 2020; Klabacka et al. 2020; Lim et al. 2020; Shaney et al. 2020; Esselstyn et al. 2021; Hinckley et al. 2022, 2023; Hinckley, Camacho‐Sanchez, et al. 2024; Sholihah et al. 2021; Arifin et al. 2022; Berman et al. 2024; Cheng and Faidi 2024).

While this region's geographical complexity provides an excellent setting to study evolution, it also poses great challenges, often leading to limitations in sampling and constraining the geographical scope of regional studies. Incomplete sampling hinders our understanding of the factors shaping diversification. High‐throughput sequencing, combined with museum genomics, overcomes historic sampling limitations (Card et al. 2021). This advancement allows for more robust phylogenomic inferences and precise divergence dating in this complex and dynamic region. By sequencing DNA from museum specimens obtained over the last century or more, museum genomics also enables the study of highly elusive, protected and/or endangered species from rarely sampled/collected remote regions, greatly enhancing sample sizes and geographic coverage (Hawkins et al. 2022).

The canopy‐dwelling Oriental Giant squirrels (*Ratufa*) represent a prime example of elusive and protected species that are currently difficult to sample in field studies. These species are highly vulnerable to forest fragmentation. In fact, three of the four extant species are catalogued by the IUCN as Near Threatened, due to significant declines driven by habitat loss and overhunting for local consumption (MacKinnon 1978; Payne 1979; Saiful and Nordin 2004; Datta and Goyal 2008; Shyam and Saikia 2012; Koprowski et al. 2016; Sengupta et al. 2023). These squirrels' strictly arboreal and forest‐dependent nature, combined with their widespread distribution throughout tropical Asian lowlands, makes studying their evolutionary history key to understanding the intricate interplay between this region's paleo‐forest distribution and regional diversification.

This iconic genus is the only extant representative of the Ratufini Tribe, a lineage sister to all other Sciuridae (Abreu et al. 2022), with fossil records tracing back to the early Miocene (e.g., †*Ratufa sylva*, †*R. maelongensis*, and †*Ratufa indet*.; Mein et al. 1990; Flynn 2003; Flynn and Wessels 2013). It contains four extant species distributed throughout the Indian subcontinent ( *R. indica* and *R. macroura* ), Indochina and West Sundaland (*R. bicolor*) and most of Sundaland ( *R. affinis* ). The Indian species ( *R. indica* and *R. macroura* ) exhibit modest geographically structured morphological variation, with four and three subspecies, respectively. In contrast, the two Indochinese and/or Sundaic species ( *R. bicolor* and *R. affinis* ) exhibit greater geographically structured morphological variation, with eleven and nine subspecies, respectively (Thorington Jr et al. 2012; Koprowski et al. 2016). These species/subspecies have primarily been described (and revised) based on pelage and/or size variation despite a lack of diagnostic discrete descriptive skull characters (Moore and Tate 1965). Remarkably, four dwarf populations have been identified on small islands, including Bunguran, Serasan, and Laut, in the Natunas ( *R. affinis* ; Thomas and Hartert 1894, 1895; Bonhote 1900), and Con Dao, in southern Vietnam (*R. bicolor*; Kloss 1920). The evolutionary relationships among these species are not well understood due to a lack of support in key nodes in sciurid‐wide phylogenies (Bahuguna and Singh 2019; Menéndez et al. 2021). Furthermore, challenges in trapping these high‐canopy species, their protected status in many countries, and the concomitant difficulties in obtaining fresh tissue samples have hindered the study of their evolutionary history at the intraspecific level.

*Ratufa bicolor* and *R. affinis* are sympatric in the Malay Peninsula and Sumatra. However, *R. bicolor* is found in the drier, and more seasonal evergreen forests of Java and Indochina, while *R. affinis* is restricted to the wetter rainforests of Borneo. Although their ecological requirements are not well understood, their distribution suggests distinct yet overlapping habitat requirements. This is evidenced by their sympatric distribution in areas with intermediate rainfall ranges and allopatry outside of such ranges (Phillipps and Phillipps 2016). Potential differences in resilience to rainfall seasonality and associated ecological changes are likely reflected in contrasting and asynchronous diversification patterns among populations. During drier glacial periods, the presumed wet‐adapted species *R. affinis* is expected to show higher levels of population divergence due to (a) limited dispersal across seasonal forests and (b) smaller populations leading to increased genetic drift and isolation during rainforest fragmentation. Conversely, during wetter periods, the presumed dry‐adapted species *R. bicolor* would likely exhibit higher levels of population divergence. Understanding how environmental changes interact with species traits to shape natural populations is crucial for predicting their responses to ongoing global changes.

Here we use genomic data, including complete mitochondrial genomes and a panel of ~4000 nuclear ultraconserved elements (UCEs), from all species of *Ratufa* (and all subspecies of *R. affinis* and *R. bicolor* ), integrated with ecological niche modelling to: (a) elucidate paleo‐forest dynamics and regional evolutionary mechanisms potentially fostering speciation in tropical Asia; (b) evaluate whether the partially sympatric *R. affinis* and *R. bicolor* exhibit varying levels of resilience to forest seasonality and, if so, determine if these differences have contributed to distinct evolutionary trajectories; (c) test whether dwarfism has evolved convergently and rapidly within this genus, or if it stems from a single ancient common ancestor.

## 2 Material and Methods

### 2.1 Molecular Sample Collection

We sampled remains of muscular tissue adherent to skulls, or when unavailable, broken and loose turbinate bones/dry skin/toe clips, from 109 historic museum specimens housed in the Smithsonian National Museum of Natural History (USNM; *n* = 81), American Museum of Natural History (AMNH; *n* = 16), Field Museum of Natural History (FMNH; *n* = 8), Academy of Natural Sciences of Drexel University (ANSP; *n* = 4), and obtained one frozen blood sample from Omaha's Henry Doorly Zoo and Aquarium (OHDZA hereafter, Table S1). We sampled all four extant species of *Ratufa*, and 25 of its 27 recognised subspecies (Koprowski et al. 2016). Only the subspecies *R. indica dealbata* and *R. indica centralis* were not included. For outgroup and/or fossil calibration purposes of the UCE analyses, we included samples from three other Sciuridae subfamilies: **Callosciurinae** (*Callosciurus erythraeus*, AMNH272458; *Dremomys rufigenis*, AMNH272165), **Sciurinae** (*Hylopetes spadiceus*, ROM107782), and **Xerinae** (*Xerus rutilus*, USNM601526), all of which we sequenced and which are sister to Ratufinae. In addition, we included *Aplodontia rufa* (GCA_004027875), downloaded from GenBank, which represents the closest extant relative of Sciuridae. Finally, we also downloaded a mitochondrial genome sequence of *Ratufa bicolor* (KP708709). In total, 110 ingroup samples and four outgroup samples were included in this study. A complete list of specimens, along with catalogue and geographic data and other relevant information is provided in Table S1.

### 2.2 DNA Extraction and Library Preparation

DNA extractions of historic samples were performed using the QIAamp DNA Mini Kit (Qiagen Inc.) following Hawkins et al. (2022) but with a double final elution in 30 μl instead of 50 μl, which yields a higher DNA concentration. These extractions were processed in an isolated historic DNA facility at the Smithsonian Museum Support Center, following strict protocols to avoid contamination. DNA was extracted from the frozen OHDZA blood sample using a Zymo DNA/RNA Viral MagBead Kit (#R2140) following the manufacturer's protocol for blood. Extract concentration was assessed from a Qubit Fluorometer, and a total of 55 μL of DNA at 0.56 ng/μL was sheared with a Covaris ME220 sonicator using the pre‐programmed shearing protocol for 200 bp fragments. Sheared DNA was visualised on agarose gel and a TapeStation to confirm the resulting fragment size around 250–300 bp.

Dual indexed sequencing libraries were generated with an Illumina Library Preparation—Kapa Biosystems Kit (Catalogue #KK8232) for USNM samples, SRSLY NGS Library Preparation Kit (CLARETBIO) for AMNH and FMNH samples and important USNM samples (holotypes/topotypes) that failed/performed poorly on the first attempt with the Kapa Biosystems Kit, and a KAPA HyperPrep Kit (Catalogue #KK8502) for the OHDZA blood sample. DNA extractions processed with the SRSLY kit were quantified and generally concentrated with KAPA beads before library preparation. We followed the manufacturer instructions for KAPA (but 18 cycles of amplification) or a modification of the ‘single strand’ SRSLY NGS Library Prep Kit protocol described in Troll et al. (2019) with half‐volume reactions to reduce costs.

### 2.3 UCE and Mitochondrial Genome Capture and Sequencing

Libraries were not pooled for enrichment; instead, probes were diluted with water to reduce costs (1:1 dilution for UCEs and 1:4.5 dilution for mitogenomes). UCE enrichments were performed using the myBaits UCE Tetrapods 5Kv1 kit (Arbor Biosciences), containing a probe set of around 5000 UCE loci. Mitogenome enrichments were performed using a custom myBaits kit (Arbor Biosciences). The species *Ratufa bicolor* was used as a reference to design these probes. We followed the MyBaits High Sensitivity protocol with a single round of enrichment. Hybridization temperatures were set at 63°C. Post‐capture amplifications were performed using KAPA HiFi Hotstart ReadyMix (Roche Sequencing), following the MyBaits established protocol profile with 10 and 18–25 cycles of amplification for modern and historic samples, respectively.

A 1.8× SPRI magnetic beads cleanup was performed. Cleaned amplification products were quantified using a Qubit 2.0 fluorometer. Equimolar pooling of samples was based on the concentration (ng/μL) and the average size (bp) of amplified fragments. After pooling, qPCR was conducted on the samples with replication and dilution. The average fragment size (101 bp) was determined from a final TapeStation electropherogram. The qPCR was performed using an ABI ViiA7 with the KAPA Library Quantification Kit (#KK4824). Dual‐indexed libraries were sequenced in five different NovaSeq S4 or X Plus runs (using 2 × 100 PE) at the Oklahoma Medical Research Foundation NGS Core.

### 2.4 UCE Data Processing, Assembly and Taxon Filtering

We processed the UCE data using the PHYLUCE 1.7.3 pipeline (Faircloth et al. 2012; Faircloth 2016), adapted for the Smithsonian Institution High Performance Cluster (SI‐HPC; https://doi.org/10.25572/SIHPC). Adapter contamination and low‐quality bases were removed using Illumiprocessor 2.10 (Bolger et al. 2014; Faircloth 2013). The reads were assembled into contigs with Spades 3.15.5, and these contigs were then matched to the uce‐5 k‐probe‐set (Faircloth et al. 2012). Samples with fewer than 1000 successfully enriched UCEs were excluded from downstream analyses. These samples consistently clustered together, irrespective of their taxonomic identification, and exhibited abnormally long branch lengths in preliminary phylogenetic analyses, possibly due to a combination of DNA degradation, missing data and/or contamination. Five samples that passed the initial 1000‐UCE threshold were excluded due to contamination ( *Homo sapiens* or Mustelidae in BLAST search; USNM 239285, 258881, 357266, 113162 and 142332) or a high degree of missing data (76%–79%; USNM 104628, 198791). None of these excluded samples with an early‐branching position represented singleton taxa nor exhibited distinct phenotypes to sequenced conspecifics upon examination. A total of 46 out of the 110 samples enriched for UCEs (42%) were suitable for inclusion in downstream genomic analyses.

Three distinct datasets were preliminarily generated using the *phyluce_align_get_only_loci_with_min_taxa* function. These included UCEs enriched in at least 60% (3692 UCEs), 75% (3224 UCEs), and 90% (796 UCEs) of samples. We kept the 75% taxon representativeness matrix for downstream analyses, as it provided a good balance between the number of loci and the amount of missing data. Poorly aligned sequences were removed from the concatenated alignment with SPRUCEUP and a manual cutoff of 0.06 for all samples (Borowiec 2019). After exploratory testing with IQ‐TREE, this combination produced the most balanced tradeoff, effectively reducing extremely long branches while preserving recent evolutionary lineages with shallow divergences. Nine low‐quality samples with persistent, abnormally long branches were excluded: AMNH163517, FMNH37951, USNM 254740, 355785, 277615, 257720, 113134, 114361, 357269. These specimens and other sequences excluded in downstream analyses did not represent singleton taxa and each excluded specimen (pelage and skull) was examined and found to match specimens that remained in the reduced genetic data.

### 2.5 Mitogenome Data Processing and Assembly

Reads were demultiplexed from the sequencing cores, and adaptor removal and quality trimming were performed with Trimmomatic (Bolger et al. 2014) with the sliding window parameter set to 5:20, read minimum length parameter to 30 bp (NovaSeq modern and all historic sample libraries), and leading and trailing to 5 for the remaining samples. Quality‐trimmed reads were mapped to the mitogenome of *Ratufa bicolor* (KF575124) with BWA‐MEM (Li 2013). The output BAM files were sorted, PCR duplicates were removed with SAMtools (Li et al. 2009), and libraries from the same specimens were merged. BAM files were imported to GENEIOUS PRIME 2023.0.4 where consensus sequences were called with minimum 3× coverage and 75% threshold. Visual inspection of BAM files suggested that samples of *Ratufa affinis* , *R. macroura* and *R. indica* were too divergent from the *R. bicolor* reference (KF575124) given that reads were not mapped to the most variable regions of the mitogenome, yielding an “inverse skyline pattern”. To overcome this issue and improve read mapping efficiency, we generated a “de novo” assembly with SPADES 3.15.5 (Bankevich et al. 2012) for each of these species (*R. afffinis*‐USNM124868; *R. indica* ‐FMNH82909; *R. macroura* ‐FMNH96322). We employed a range of k‐mer sizes, including 21, 33, 55 and 77. We imported the assembly contigs to GENEIOUS PRIME, mapped these to KF575124 and generated a consensus by majority. We then used these consensus sequences as references to map the reads of all samples belonging to *R. affinis, R. indica*, and *R. macroura*. West Sunda *R. affinis* and nominotypical *R. macroura* read mapping was still suboptimal, so we generated two additional “de novo” assemblies as references for these taxa (FMNH8289 and AMNH240914) following the same described procedure. We prioritised higher coverage samples for the “de novo” assemblies. References for *Ratufa macroura* were chimeric; the selected reference still had data gaps that were filled by sequence data of its closest relative, *Ratufa indica* .

### 2.6 UCE and Mitogenome Phylogenetic Analyses

We performed phylogenetic inferences on the UCE data matrix using a supermatrix (Maximum Likelihood [ML]) and two coalescent‐based approaches: a two‐step summary coalescent method (inferring the species tree from individual gene trees) and a site‐based method. We estimated maximum‐likelihood trees for the UCE concatenated dataset (47 taxa, 1,091,360 total sites, and 7.24% of missing data) using IQ‐TREE 2.3.1 (Minh et al. 2020). We obtained branch supports using the ultrafast bootstrap method (UFboot; Hoang et al. 2018) with 1000 replicates. We followed three partition approaches for this analysis. First, we ran an unpartitioned analysis with all concatenated loci and with the GTR + F + G4 model of substitution. We chose this model for all loci without conducting model testing, as more complex models do not adversely affect phylogenetic estimations, even if simpler models might be preferable for certain loci (Abadi et al. 2019; Abreu et al. 2022). Secondly, analyses were performed with the dataset partitioned by locus and with the GTR + F + G4 model of substitution set for each locus. Finally, we utilised the Sliding‐Window Site Characteristics based on entropy approach (SWSC‐EN) to partition UCE loci into right flank, core, and left flank regions (Tagliacollo and Lanfear 2018). This method is optimised to UCEs' heterogeneous structure, characterised by conserved core regions and flanking regions with increasing variability. After running SWSC‐EN, two separate analyses were performed with the output partitions: unmerged partitions and with the GTR + F + G4 model of substitution, and IQ‐TREE's “automerge” option and the rclusterf algorithm, which automatically merges partitions that have similar evolutionary models. The resulting best‐fit partitioning scheme (SWSC‐EN‐GTR + F + G4: −1475523.37) included 7907 partitions and had a higher log likelihood and lower BIC than alternative partitioning schemes that we tested (SWSC‐EN‐automerge: lnL = −1505680.665, BIC = 3023459.0440; By Locus: lnL = −1773830.857, BIC = 4003784.1859; Unpartitioned: lnL = −1800273.433, BIC = 3601855.6434). Branch support and topology were almost identical among different ML analyses, suggesting robust phylogenetic inference.

Coalescent species tree analyses were conducted using SVDquartets (Chifman and Kubatko 2014), implemented in PAUP* v4a168 (Swofford and Sullivan 2003), and ASTRAL‐III v.5.7.8 (Rabiee et al. 2019). Input files for SVDquartets were generated with the uce2speciestree.rb script from all phyluce UCE loci alignments that were included in the IQ‐TREE analysis (https://github.com/campanam/uce2speciestree). ASTRAL‐III v.5.7.8 generates a species tree by summarising and weighting input gene trees equally. We first generated a maximum‐likelihood gene tree for each UCE locus with RAxML. However, ca. 25% of UCE loci (798) lacked parsimony‐informative sites, and many others contained only weak phylogenetic signal, rendering poorly resolved gene trees. To overcome these issues, we sorted the UCE loci by the number of parsimony‐informative sites using the *phyluce_align_get_informative_sites* script and reduced the dataset to the top quartile of loci, as in Mills et al. (2023). We then collapsed very‐low‐support branches (Bootstrap < 30) with Newick Utilities in each of the 806 UCE loci in the reduced dataset to improve accuracy (Zhang et al. 2017; Rodríguez‐Machado et al. 2024). We retained individuals as operational taxonomic units (OTUs) rather than grouping them by species (i.e., we opted not to use the ‐a flag). Missing data in input gene trees can affect species tree inference with ASTRAL, leading to reduced concordance, resolution, and biased relationships. Upon visualisation of a preliminary analysis, we realised that eight samples with high levels of missing data in the concatenated alignment and relatively longer branches in the ML analysis had potentially artificial early‐branching positions in the ASTRAL‐III tree ( *R. affinis* : FMNH 8289, USNM 115528, 122879, 123124, 143351, 145372, 488101; *R. bicolor* : USNM300026). Thus, these samples were excluded with APE (Paradis and Schliep 2019) from the input gene trees with the “drop.tip” function and ASTRAL‐III was rerun with the pruned trees which had a total of 39 terminals (Table S2).

Maximum likelihood (ML) phylogenetic inference was conducted with IQ‐TREE 2.3.1 on the mitochondrial genome dataset (Minh et al. 2020). Substitution model selection and the best‐partition scheme were carried out with IQ‐TREE's ModelFinder implementation (Kalyaanamoorthy et al. 2017). The input 53 partitions were merged into 10 partitions, the FreeRate heterogeneity model was considered, a relaxed clustering of 10% was selected, and each partition was allowed to have its own evolutionary rate (−m MFP + MERGE; Appendix). ML tree reconstruction was run following the best‐partition model. Edge‐linked with proportional branch lengths (−spp) and unlinked‐edge (−sp) partition models were run, with the former being selected due to its lower BIC scores (Appendix). Ultrafast bootstrap support (UFBoot) was computed with 1000 replicates.

### 2.7 Divergence Dating

We filtered input alignments for this analysis to minimise the amount of missing data and DNA damage artefacts still present after running Spruceup. Damage artefacts can inflate branch lengths, biasing divergence estimates. Therefore, we kept a single sample (the one with the least missing data) for each highly supported clade in the three previous analyses and included only those loci with sequence data for all samples, resulting in a matrix of 2046 loci. Following visual inspection of the alignment, we realised that gap‐filled regions and missing‐data areas are challenging for Spruceup's algorithm, particularly for the removal of very short outliers (1–3 bp). This was reflected in the presence of some insertions or single bp outliers contiguous to long stretches of Ns. To overcome this issue, we first trimmed regions with gaps with the command phyluce_align_get_trimal_trimmed_alignments_from_untrimmed. Next, we computed alignment summary stats with AMAS (Borowiec 2016) and kept for downstream analyses 210 alignments with less than 8% missing data with phyluce_align_move_align_by_conf_file (Table S2). Following concatenation, we manually inspected the alignment on GENEIOUS and replaced with Ns these apparent mutations that are adjacent to long stretches of Ns and that were most likely caused by damage or degradation of the DNA.

We harvested UCEs from the *Aplodontia rufa* (GCA004027875) NCBI genome and added the following outgroups: *Callosciurus erythraeus* AMNH272458, *Dremomys rufigenis* AMNH272165, *Hylopetes spadiceus* ROM107782, and *Xerus rutilus* USNM601526. These outgroups were selected, in part, to enable fossil calibration.

We estimated divergence times with BEAST 2.6.7 (Bouckaert et al. 2019). To calibrate the timetree, we followed the methodology outlined by Mills et al. (2023), using *Aplodontia rufa* as the outgroup, and constraining the root of the tree to a minimum of 40.4 million years ago (Ma), based on †*Douglassciurus oaxacensis*, the oldest known sciurid fossil (Ferrusquia‐Villafranca et al. 2018). We set a maximum age of 57 Ma, based on †*Acritoparamys atavus*, the oldest stem member of Sciuromorpha (Ivy 1990; Korth 1994; NOW database). Similar constraints have been applied in other divergence dating studies (Meredith et al. 2011; Menéndez et al. 2021). Two more constraints were set using hard minimum bounds and soft upper bounds using a lognormal prior, as suggested by Parham et al. (2011). In line with Menéndez et al. (2021), we calibrated the MRCA Xerinae + Callosciurinae using the oldest Xerinae stem fossil, *Palaeosciurus goti*, dated to 32.6 mya (Vianey‐Liaud 1974). Additionally, we followed the approach of Hinckley et al. (2020, 2023) by using a fossil of *Callosciurus* sp. (specimen number: YGSP 21682). This specimen has been dated through paleomagnetic correlation to 14 mya (Flynn 2003) to calibrate the *Callosciurus‐Dremomys* clade MRCA. The oldest known *Ratufa* fossil (Flynn and Wessels 2013) was not used for timetree calibration because older fossils were available to calibrate a shallower node representing the most recent common ancestor (MRCA) of Xerinae and Callosciurinae. Calibrating both nodes (*Ratufa* vs. other squirrels and Sciurinae vs. Xerinae + Callosciurinae) would not make sense since the oldest node would be calibrated with a younger fossil than the youngest node, and vice versa. We did not use *Plesiarctomys spectabilis* to calibrate *Sciurus* and other sciurids, as done in Mills et al. (2023), because it is currently classified as a member of Ischyromyidae, not Sciuridae, according to the NOW database of fossil mammals (2024, https://nowdatabase.org/now/database/).

We specified a birth–death process to account for both speciation and extinction, since we calibrated our phylogeny with fossils representing extinct species. We conducted eight runs with different calibration (single vs. double vs. triple calibration), substitution (HKY + Gamma 4 and empirical frequencies vs. bModelstest [all reversible models]), and clock model combinations (strict clock vs. optimised relaxed clock [ORC] vs. Uncorrelated Lognormal Relaxed Clock [UCLN]). To determine the best‐fitting model combination, we performed Bayes Factor tests using the marginal likelihoods of all runs that exhibited high ESS values in TRACER 1.7.2 (Rambaut et al. 2018). We compared models progressively, starting from the simplest and moving towards more complex combinations. Only some parameters of the UCLD model did not converge, suggesting a poor fit for this model. The bmodeltest+ORC with three calibrations was strongly supported as the model with the best fit in all comparisons (Appendix). Three independent runs of Markov chains for Monte Carlo (MCMC) simulations were run on this highly supported model for 100,000,000 generations, with parameters and trees sampled every 5000 generations and a burnin of 10,000,000. An additional first 10% of sampled trees of each run were discarded as burn‐in, before all three independent runs were combined with LogCombiner and a maximum credibility consensus tree of the combined trees was generated with TreeAnnotator (Bouckaert et al. 2019).

### 2.8 Genealogical Concordance Tests

We computed gene and site concordance factors (gCF and sCF) with IQ‐TREE 2.3.1 to gain insights into gene tree discordance, hard polytomies, incomplete lineage sorting, and introgression in certain clades with poor nodal support. gCF is defined as the percentage of “decisive” gene trees that contain a given branch. sCF, on the other hand, represents the percentage of decisive alignment sites that support a branch in the reference tree. In this context, “decisive” gene trees or sites are those that contain enough information (i.e., the relevant taxa and resolution) to potentially support or reject a given branch in the species tree. This novel sCF measure is especially valuable when individual gene alignments provide limited information, resulting in uncertain gene trees. We followed the first alignment filtering steps as in the BEAST analysis, by selecting a single representative from each major lineage, prioritising those with the least missing data, and only keeping those loci in which all input samples were represented, leading to a matrix of 2046 loci. This approach aimed to maximise the number of loci included in the analysis while minimising incomplete gene trees, which could otherwise bias the results (Table S2). We computed gene tree inference with IQ‐TREE and the GTR + G4 substitution model. We ran the gCF analysis twice, with and without contracting low‐support branches (UltraBP < 70) of input gene trees, to determine the impact of collapsing these branches on the results.

### 2.9 Ecological Niche Modelling

#### 2.9.1 Occurrences Data

We gathered data on 243 geographic occurrences for *R. affinis* and 749 for *R. bicolor*. These localities were obtained from examined specimens from USNM, ANSP, AMNH, FMNH, and NRM (Swedish Museum of Natural History), as well as GBIF verified records with pictures that we examined—including iNaturalist, Observation.org, biodiversity.bt, indiabiodiversity.org [GBIF.org (25 July 2023) GBIF Occurrence Download (https://www.gbif.org/occurrence/download/0113869‐230530130749713)]. Additionally, we included two unverified iNaturalist records with pictures that were downloaded from this platform, and were carefully examined, and verified (Table S3).

To reduce spatial sampling bias in the occurrence, which has been shown to improve ENM predictions (Boria et al. 2014), we used the R package *spThin* 0.2.0 (Aiello‐Lammens et al. 2015) to spatially thin the occurrence localities by applying a 10 km minimum distance.

#### 2.9.2 Environmental Variables

We used 14 bioclimatic variables (bio01, bio04, bio08–bio19) from multiple climate datasets. Based on temperature and precipitation, bioclimatic variables summarise monthly averages, seasonal patterns, and extreme conditions based on monthly data, and they have been widely used and accepted in species distribution modelling (Booth et al. 2014). Variables were obtained from three sources: WorldClim v2.0 at 30 arc‐seconds resolution for the present (Fick and Hijmans 2017), CHELSA at 2.5‐min resolution for the Last Glacial Maximum (LGM) (Karger et al. 2017), and Paleoclim.org at 2.5‐min resolution for the mid‐Pliocene Warm Period (mPWP; ~3.264–3.025 Ma) (Hill 2015; Brown et al. 2018). Due to the unavailability of maximum and minimum temperature variables when building the Pliocene dataset, certain temperature‐related predictors, such as bio05 and bio06, were omitted from our analyses. The full set of available variables was used as input for model training, with no need to remove highly correlated variables due to the algorithm used in this study (Feng et al. 2019).

#### 2.9.3 Model Training, Evaluation and Transfer

Model training was conducted using the Maximum Entropy algorithm implemented via the *maxent* package (v0.1.4; Phillips et al. 2017). The training area was defined by 20 km buffers around ecoregions (Olson et al. 2001) with at least one occurrence, which could potentially represent geographical barriers that historically constrain species distribution (Barve et al. 2011; Rojas‐Soto et al. 2024). The *ENMeval* package (v2.0.4; Kass et al. 2021) used spatial block partitioning to explore model complexity. Various combinations of feature classes (Linear, Linear‐Quadratic, Quadratic, Linear‐Quadratic‐Product) and regularisation multipliers (ranging from 0.1 to 1 in increments of 0.1, and from 1.5 to 5 in increments of 0.5) were tested. Model selection was based on the Akaike Information Criterion corrected for small sample sizes (AICc; Warren and Seifert 2011). Additional metrics, including the omission rate at the 10th percentile (OR.10P), the area under the receiver operating characteristic curve (AUC), and the continuous Boyce index (CBI), were assessed to validate model performance. A custom clamping approach (Kass et al. 2021) was applied to ensure appropriate extrapolation when transferring the models to past scenarios. Maps of the predicted distributions were obtained using a binary threshold based on the 10th percentile training presence threshold. Variable importance was assessed by randomly permuting the training values 100 times and measuring the resulting decrease in AUC values; a greater decrease indicates higher variable importance (i.e., permutation importance; Phillips 2006). The maps were created using the *ggplot2* (v3.5.1; Wickham 2016), *ggspatial* (v1.1.9; Dunnington 2020), and *tidyterra* (v0.6.1; Hernangómez 2023) packages in R.

#### 2.9.4 Niche Overlap and Similarity

Additionally, tests of niche overlap and similarity were performed between the two *Ratufa* complexes using the R package *ecospat* (v4.1; Di Cola et al. 2017). Niche overlap was quantified using Schoener's D, a quantitative index that measures the degree to which two taxa (in this case, the two *Ratufa* complexes) overlap in environmental space (Warren et al. 2008). An index value of 1 indicates complete overlap, while a value of 0 indicates no overlap. The similarity test compares the niche overlap against a null distribution of overlap values generated by randomising the background environmental values (Broennimann et al. 2012). A *p*‐value lower than 0.05 indicates that the two niches are more similar than expected by chance. The same occurrence and background data used for model training were applied for these two analyses.

### 2.10 Morphological Data Collection and Analysis

Pelage variation was examined in all specimens from USNM, ANSP, AMNH, FMNH, and NRM, and GBIF verified records with pictures shown in Tables S1, S3 and S4. Specimens were identified to subspecies based on their pelage characteristics according to Moore and Tate (1965) and Thorington Jr et al. (2012).

Occipitonasal (skull) length measurements, as defined in Musser et al. (2010), were collected with the aim of highlighting body size variation between standard‐sized and dwarf populations of *R. affinis* and *R. bicolor* . Fusion of presphenoid‐basisphenoid and basisphenoid‐basioccipital sutures as well as dental eruption and wear patterns were examined to estimate the age of specimens. Only adults were measured. Craniodental measurements were taken with high precision electronic digital callipers to the nearest 0.01 mm by a single observer (AH), and are shown in Table S4. We illustrated body size differentiation between standard‐sized and dwarf populations separately for *R. affinis* and *R. bicolor* through univariate plots constructed with *ggplot2* (v3.5.1; Wickham 2016).

## 3 Results

### 3.1 Molecular

Whole mitogenomes were constructed from 35, 34, 4 and 4 individuals, respectively for *R. affinis* , *R. bicolor* , *R. indica* , and *R. macroura* , generating an alignment of 15,634 total sites, 80 sequences (three were downloaded from GenBank), and 0.02% missing data used to construct a ML phylogeny (Table S1). A 42% of samples enriched for UCEs were suitable for inclusion in downstream genomic analyses. UCE data were “successfully” generated (see methods) for 23, 19, 2, and 1 individuals, respectively for *R. affinis* , *R. bicolor* , *R. indica* , and *R. macroura*. An alignment of 1,091,360 bp, 3224 loci and 47 sequences was yielded for the IQ‐TREE and SVDquartets, while 262,646 bp, 806 loci, and 39 sequences were included in the ASTRAL analyses. Finally, the BEAST and concordance‐factor analyses included 114,353 bp, 210 loci, and 8 ingroup +5 outgroup sequences and 1,222,917 bp, 2046 loci, and 8 ingroup +5 outgroup sequences, respectively (Tables S1 and S2).

**FIGURE 1:**
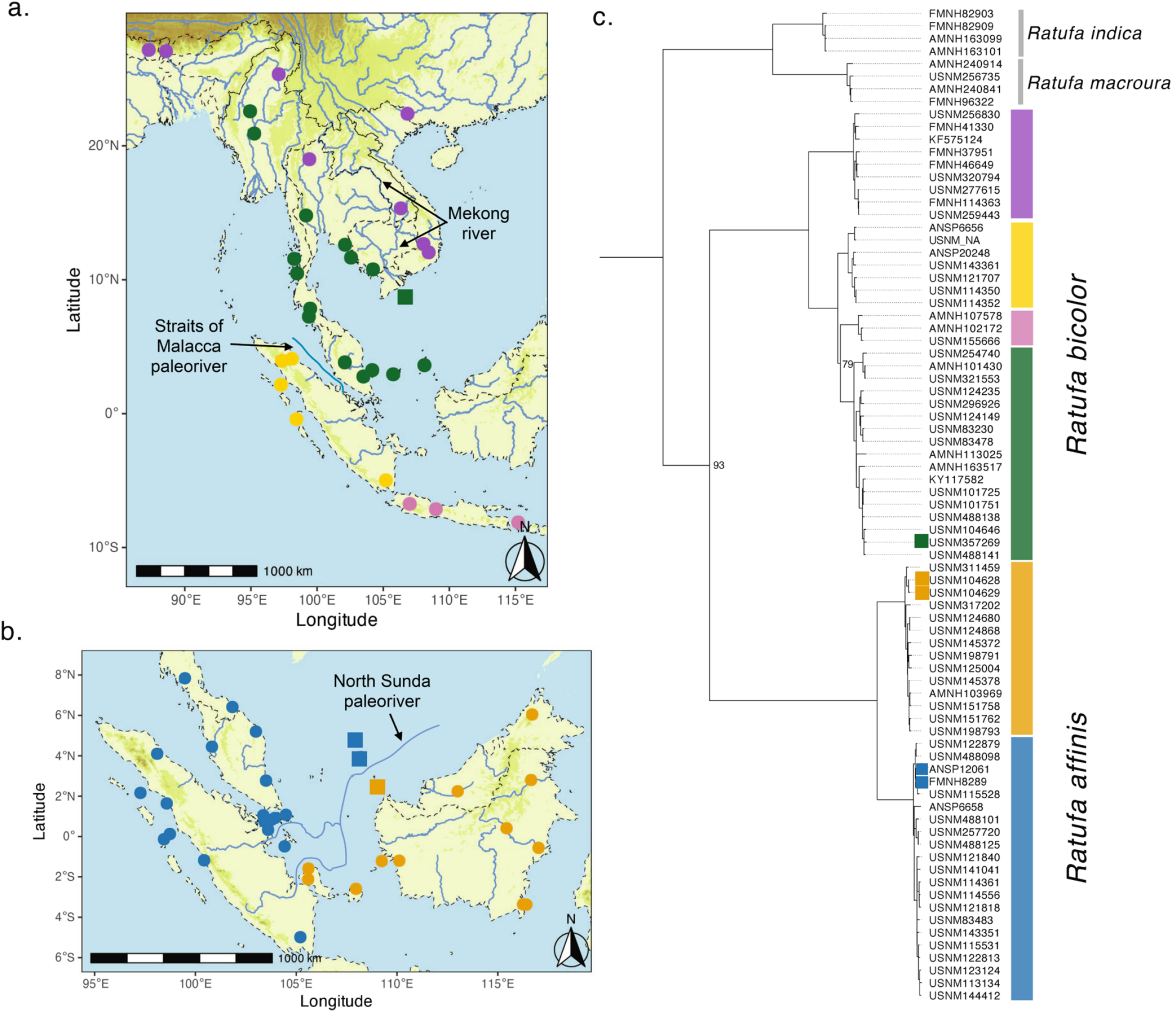
(a) Map of tropical East Asia showing sampling localities (points) with mitochondrial genome data for *Ratufa bicolor* . (b) Map of Sundaland displaying sampling localities (points) with mitochondrial genome data for *Ratufa affinis* . Different lineages are represented by distinct colours. Darker green shades indicate elevations higher than 800 m a.s.l. (c) Maximum‐likelihood consensus phylogeny of *Ratufa* species based on 80 mtDNA genomes (15,634 nt), reconstructed using IQ‐TREE 2. Ultrafast bootstrap (UB) support values are shown for only nodes with support below 95%. The geographic origin of the study species ( *R. affinis* and *R. bicolor* ) is indicated by colours on the adjacent vertical bar, corresponding to the colours used in the maps (a, b) and other figures. Tip labels represent museum catalogue numbers, other specimen identifiers, or GenBank accession numbers (see Table S1). Outgroup *Dremomys rufigenis* was used for rooting (not shown). Dwarf population localities and tree tip labels have been highlighted with a square.

**FIGURE 2:**
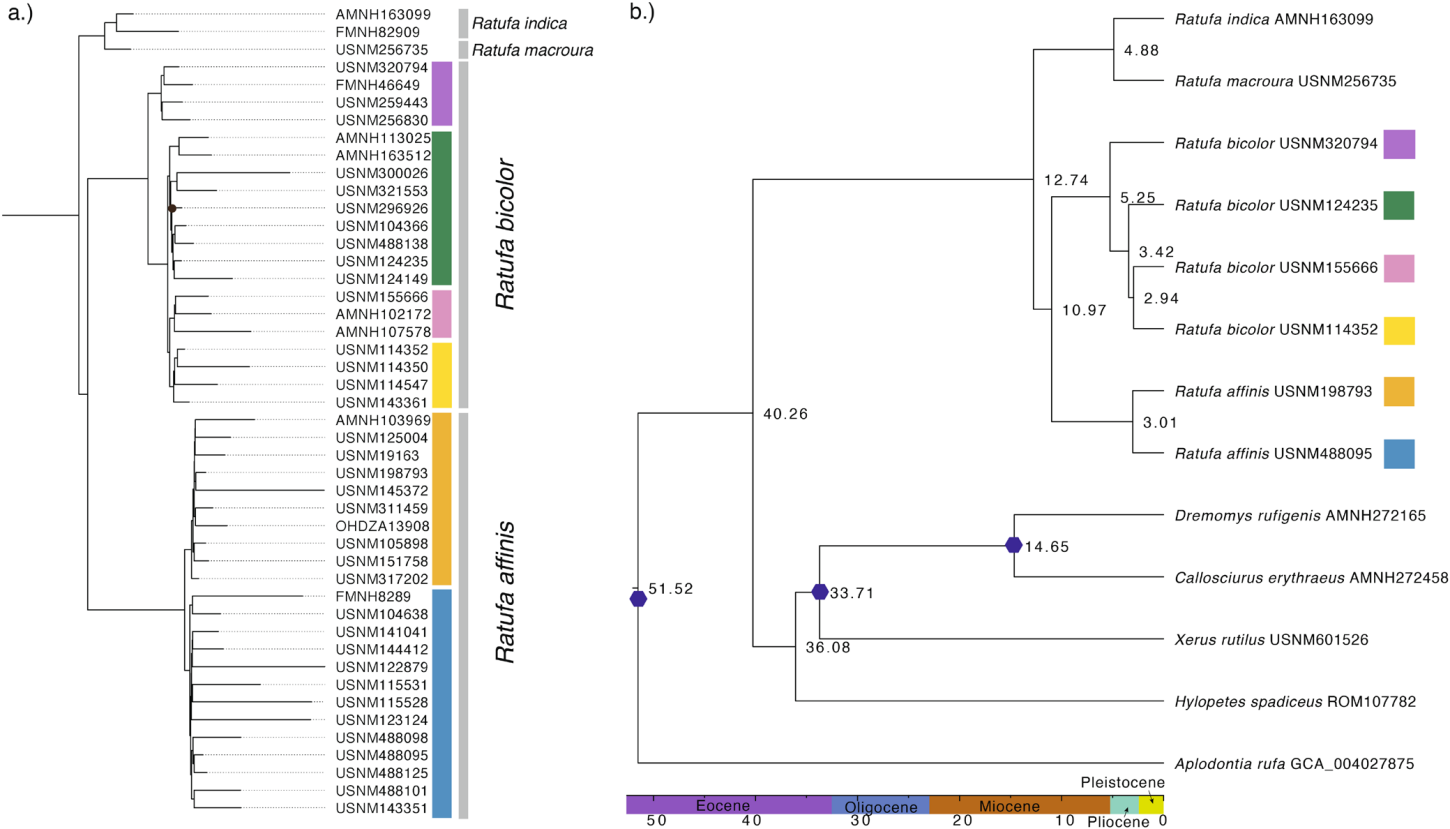
(a) Maximum‐likelihood tree estimated in IQ‐TREE from the 75% complete UCE dataset (1,091,360 bp, 3224 loci). Tip labels represent museum catalogue numbers or other specimen identifiers (see Table S1). Branch support values (UB) are highly supported (greater than 95) unless labelled as a circle. Outgroup *Dremomys rufigenis* was used for rooting (not shown). The following eight samples with high levels of missing data in the concatenated alignment have been excluded in downstream ASTRAL‐III analyses (Figure S2): *R. affinis “west”*–FMNH 8289, USNM 115528, 122879, 123124, 143351, 488101; *R. affinis “east”*–USNM 145372; *R. bicolor “south”*–USNM300026. (b) Fossil‐calibrated Bayesian maximum clade credibility tree inferred with BEAST2 from 210 UCEs with less than 8% of missing data and a 100% complete taxonset. Black hexagons correspond to fossil calibrations. Node age (in millions of years ago) is indicated next to each node; all nodes are highly supported (posterior probability = 1).

All mitogenome (Figure 1) and UCEs trees (Figure 2 and Figures S1–S5) supported the same overall topology, encompassing three clades corresponding to the recognised species *Ratufa indica* + *Ratufa macroura* , *Ratufa bicolor* , and *Ratufa affinis* . The clade including *R. indica* and *R. macroura* had a highly supported early branching position in the UCE concatenated analyses (IQTREE and BEAST) but was poorly supported in the mitogenome and UCE summary analyses (ASTRAL and SVDquartets). As expected, the mitogenome tree showed greater population‐level structure than the UCE tree, and as previously mentioned, it mirrored the UCE topology, suggesting there is little mitonuclear discordance in this genus. Within *R. bicolor* , a “north” clade from the East Himalayas and north and east Indochina was highly supported as sister to a “south” clade corresponding to south and west Indochina plus the Malay Peninsula, Sumatra, Java and Bali. The divergence between these north and south clades was greater than the divergence between recognised species *R. indica* and *R. macroura*. Within *R. affinis* , two divergent (mitogenome)/shallow (UCEs) clades were highly supported, corresponding to Borneo, Belitung, and south Natunas (East Sunda lineage), and Malay Peninsula, Sumatra, Riau and North Natunas, (West Sunda lineage). The recognised species *R. indica* and *R. macroura*, the north *R. bicolor*, and east and west Sunda *R. affinis* clades showed little structure across their ranges. Conversely, “south” *R. bicolor* populations exhibited high levels of divergence in both the mitogenome and UCE tree between three subclades representing Sumatra, Java+Bali, and the mainland with the Anambas and north Natunas. Concordance factors suggest a rapid/simultaneous cladogenesis within the “south” *R. bicolor* subclade, likely accompanied by incomplete lineage sorting (ILS) or introgression between sister lineages (gene Concordance Factor [gCF] = 3.52, gene Discordance Factor 1 [gDF1] = 2.59, gene Discordance Factor 2 [gDF2] = 3.18, Gene Discordance Factor due to Polytomy [gDFP] = 90.71; Figure S4). Both gDF1 and gDF2 are low and nearly symmetrical, which is a signature of ILS or introgression between sister lineages. In contrast, introgression between non‐sister lineages tends to produce asymmetry in the discordant topologies—for example, gDF1 or gDF2 should be much higher than the other discordant topology, reflecting excess gene flow in one direction (Hibbins and Hahn 2022). Additionally, an extremely high gDFP (90.71), indicates that gene trees are largely unresolved—as expected in rapid speciation events with ILS.

### 3.2 Species Distribution Modelling

After a 10 km spatial thinning, 121 and 325 localities were used in model training for *R. affinis* and *R. bicolor* , respectively. Model training resulted in 72 candidate models per species, which varied in the feature classes and the regularisation multiplier used. The optimal model selected for *R. affinis* used Linear‐Quadratic feature classes with a regularisation multiplier of 0.7 (ΔAIC = 0, AUC = 0.755, CBI = 0.783, OR.10P = 0.158). Meanwhile, the model chosen for *R. bicolor* used Linear‐Quadratic‐Product feature classes with a regularisation multiplier of 0.5 (ΔAIC = 0, AUC = 0.727, CBI = 0.466, OR.10P = 0.253). Regarding variable importance (Table S5), the most influential variables for *R. affinis* were precipitation seasonality (bio15; 23.6%), precipitation of the wettest month (bio13; 18.8%), and mean temperature of the wettest quarter (bio08; 14.1%). For *R. bicolor* , the top three variables were annual mean temperature (bio01; 15.5%), mean temperature of the warmest quarter (bio10; 14.0%), and temperature seasonality (bio04; 13.7%).

**FIGURE 3:**
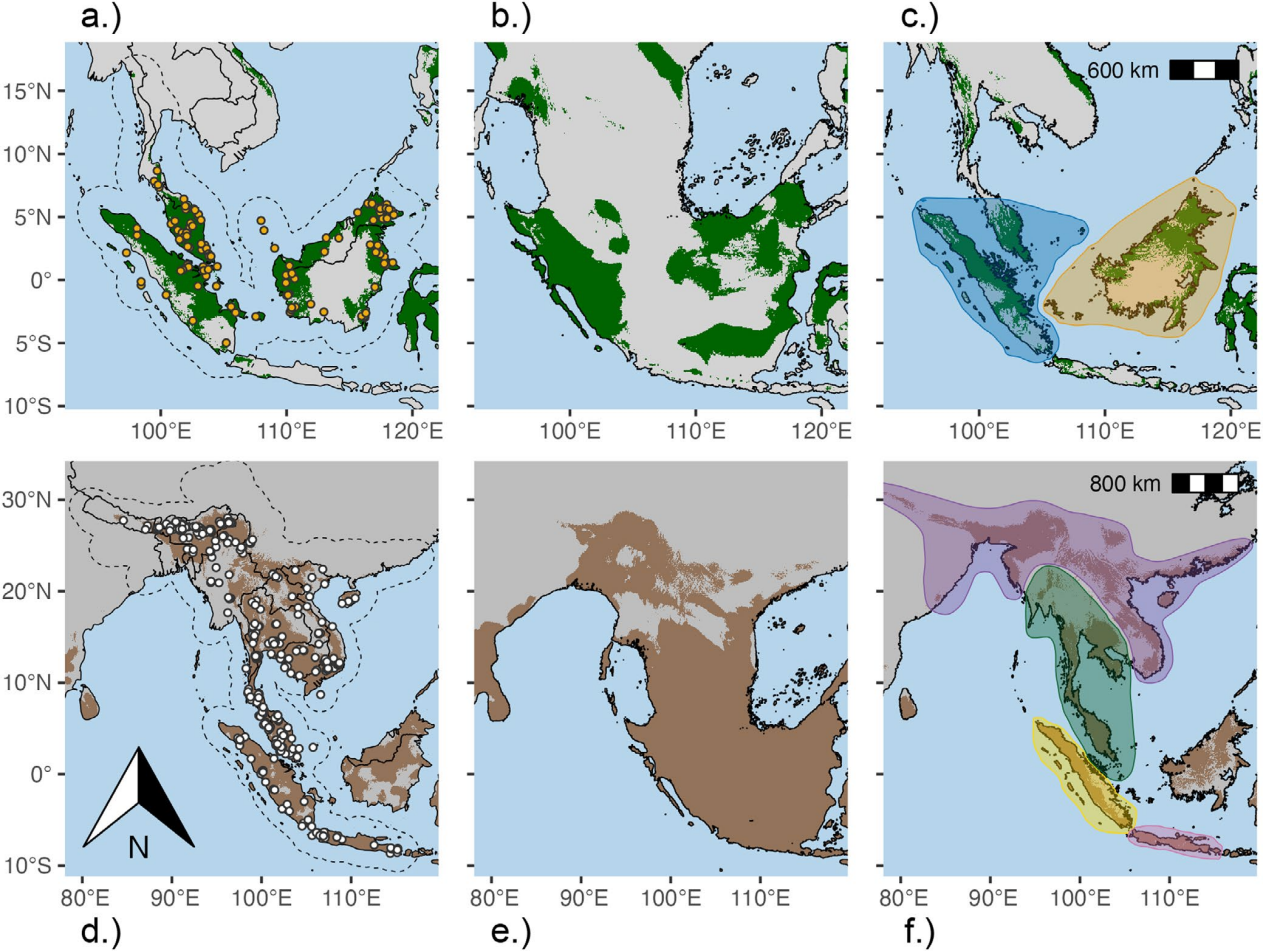
Potential geographic distribution maps of *Ratufa affinis* (a, b, c) and *R. bicolor* (d, e, f) were generated using an ecological niche model. Suitable habitats for the present (a, d), the Last Glacial Maximum (b, e), and the Pliocene (c, f) are shown in colour ( *R. affinis* in green and *R. bicolor* in brown). In contrast, unsuitable areas are shown in grey. In the present‐day predictions, the calibration area is outlined by a dashed border polygon, and the points indicate the localities used for model training. Polygons indicate the Pliocene disjunct distributions (c, f) of lineages shown in Figures 1 and 2, coloured to correspond to each lineage.

Potential distribution maps for *R. affinis* in Southeast Asia yielded varying but consistent suitability patterns across three temporal periods (Figure 3a–c). The current distribution model predicted suitable areas in well‐documented regions such as Sumatra, Borneo, and the Malay Peninsula, as well as smaller insular areas like the Bangka, Belitung and Riau Islands. Interestingly, the model does not predict suitable climates in the drier regions of Indochina, south Sumatra, and most of Java (except in a small area in the most humid western part). Similarly, the montane forests of central Borneo and the more seasonal/peatland forests of south Borneo were not predicted as suitable. The model also predicted suitable conditions beyond the species' currently reported range, notably in a large portion of Sulawesi and, to a lesser extent, fragmented areas of Java (Figure 3a). During the LGM, suitable areas were predicted to be fragmented along the Sundaland shelf, primarily covering areas where the species is currently found (e.g., Sumatra, Borneo, and the Malay Peninsula) and extending into the Java Sea region (Figure 3b). However, predicted suitability on present‐day Java Island was minimal and fragmented, with no apparent connections to other core areas. Finally, the Pliocene distribution model predicted suitability in areas similar to the present but, like during the LGM, showed a lack of connectivity between the southern Malay Peninsula and predicted distributions in mainland Indochina (Figure 3c).

For *R. bicolor* , the current potential distribution model predicted suitable climates primarily in Java, Sumatra, the Malay Peninsula, and parts of Indochina (Figure 3d). Notably, the model does not predict suitable climates in the drier regions of the Upper Mekong Valley and the Irrawaddy River basin. The predicted distribution extends northward to the Himalayan foothills, with suitable areas also identified in Nepal, Bangladesh, India, and Myanmar. Intriguingly, the model predicts suitable climates in Borneo, Sulawesi, and Sri Lanka as well. During the Last Glacial Maximum (LGM), the model projects a broad suitability area across the entire Sundaland, including Borneo (Figure 3e). In contrast, the Pliocene model shows a geographic extent of suitability similar to the present distribution, though with a somewhat reduced overall predicted range (Figure 3f). Analysis of niche overlap between taxa revealed a Schoener's D value of 0.298, suggesting moderate differentiation in their environmental niches (Figure S6). Despite this, a similarity test indicated that the observed niche overlap was not significantly greater than would be expected by chance (*p* = 0.059).

### 3.3 Morphological

Univariate plots support body size differentiation between dwarf populations and their standard‐sized relatives in the “west” and “east” lineages of *R. affinis* and in the “south” lineage of *R. bicolor* (Figure S7). Furthermore, skull size ranges do not overlap between dwarf island populations and their closest relatives (Figure S7).

## 4 Discussion

As global biodiversity faces unprecedented threats, accurately understanding the evolutionary mechanisms that foster speciation in hyperdiverse regions is increasingly important for guiding conservation efforts and predicting the impacts of climate change. Understanding speciation allows us to not only protect existing species but also the evolutionary processes that generate and maintain biodiversity (Santini et al. 2012; Zhang et al. 2019). In this research, we obtained genomic and distribution data from museum specimens and citizen science‐verified records to look at the diversification of canopy‐dwelling and forest‐dependent *Ratufa* squirrels. This integrated approach enabled us to infer paleo‐forest dynamics and uncover regional evolutionary mechanisms that potentially foster speciation in tropical Asia. By leveraging museum specimens, this study boasts one of the most comprehensive geographic coverages among recent genomic studies in Southeast Asia, providing a uniquely detailed perspective on speciation processes in this region.

Despite overlapping geographic distributions in Sumatra and the Malay Peninsula, *R. bicolor* and *R. affinis* exhibit low to moderate climatic niche overlap (Schoener's D = 0.298). However, a niche similarity test indicated that this overlap was not significantly greater than expected under a null model (*p* = 0.059). This suggests that, although the taxa share some environmental space, their climatic niches are not more similar than would be expected by chance. Notably, *R. bicolor* appears to exhibit broader climatic niche breadth (Figure S6) and greater tolerance to seasonal variation compared to *R. affinis* . This is highlighted in Sumatra, where *R. bicolor* is distributed throughout the island, occurring in more seasonal forest than *R. affinis* . (Figure 3). The contrasting greater suitability and model expansion of *R. bicolor* across Sundaland during the drier and colder LGM in comparison to a more restricted and fragmented predicted distribution in *R. affinis*, is also in line with this hypothesis. Interestingly, during the LGM the Sunda shelf was exposed, and the model predicts suitable climates in Borneo for *R. bicolor*, although this species is not presently found there (Husson et al. 2020). This suggests additional processes such as ecological adaptation and interspecific competition, may have shaped the evolutionary history of these two lineages. If there was physical and ecological connectivity for *R. bicolor* across Sundaland, it is possible that *R. affinis* outcompeted it in Borneo's humid rainforests, particularly during warmer interglacial periods. Alternatively, or in combination, *R. affinis* 's smaller size might have made it better adapted to warmer and perhumid periods. This scenario mirrors the island‐wide extinctions in Borneo of the savanna‐adapted tiger and Javan rhinoceros, particularly the latter, where its rainforest‐adapted relative, the Sumatran rhinoceros, survived until recently (Cranbrook 2010). Conversely, *R. affinis* might have had a greater dispersal capability than *R. bicolor* across potential physical or ecological barriers such as the North Sunda Paleoriver (discussed below).

The earliest speciation events in *Ratufa*, as inferred from this study, date to the Middle Miocene. Several processes likely contributed to habitat (forest) fragmentation and allopatric speciation of the most recent common ancestor (MRCA) of *Ratufa* (~13 million years ago [mya]) and of *R. bicolor* and *R. affinis* (~11 mya). First, global climate cooling from the Mid‐Miocene (around 15 mya) led to increased aridity and a contraction of evergreen and rainforests, which were likely at their greatest extent during the Middle Miocene climatic optimum (MMCO; Favre et al. 2015; Zhu and Tan 2024). This expanded extent of evergreen forests was driven not only by globally higher temperatures but also by a previous weakening of the winter monsoon during the Early Miocene and a strengthening of the Eastern Asian Summer Monsoon (EASM) during the MMCO (Li et al. 2009; Hui et al. 2023). This shift resulted in a distinctly more humid climate, since precipitation during this period reached approximately 860 mm, nearly double the present value of ~450 mm (Hui et al. 2023). Furthermore, the continued uplift of the QTP and the Himalayas, combined with global cooling, likely triggered the onset of the modern South Asian Summer Monsoon and further enhanced the EASM wind systems during the late Middle Miocene, around 13 mya (Wan et al. 2007; Gupta et al. 2015; Farnsworth et al. 2019; Zhu and Tan 2024). These “super‐monsoon conditions” align with evidence from palynological and paleoclimate records (Farnsworth et al. 2019). This increased seasonality in this region and global cooling potentially contributed to evergreen/rainforest contraction and the almost simultaneous fragmentation of the MRCA of *Ratufa* in three habitat refugia (MRCA of *R. indica* + *R. macroura* , *R. bicolor* , and *R. affinis* ). The isolation between the MRCAs of the Indian subcontinent and East Asia species is both spatially and temporally congruent with the disruption of a dispersal corridor between these regions (Klaus et al. 2016). According to a comparative phylogeography study, connectivity peaked during the Middle Miocene, coinciding with the MMCO, and decreased after 14 Ma, when drier environmental conditions developed in northern India, in the Indo‐Gangetic plain, creating a dispersal barrier for evergreen forest taxa (Klaus et al. 2016; Morley 2018). Similarly, the estimated divergence among *R. bicolor* and *R. affinis* coincides with a period around 10.5 mya, when the lowest pre‐Pliocene sea levels might have promoted dispersal across a partially exposed Sunda shelf, as hypothesized for colugos (Mason et al. 2019). Conversely, cooling trends might have also promoted vicariance in two rainforest pockets. Interestingly, these speciation events in *Ratufa* are synchronous with a pronounced increase in vicariant events in mammalian lineages of Asian tropical forests (Feijó et al. 2022) and with the diversification of other sympatric rainforest/evergreen forest taxa such as angiosperms, stream toads, pangolins, horseshoe bats, colugos, and tree squirrels (Grismer et al. 2017; Kong et al. 2017, 2022; Mason et al. 2019; Guo et al. 2021, 2023; Gu et al. 2023; Hinckley et al. 2020, 2023).

Continued global cooling and uplift intensification of the Himalayas and Tibetan Plateau peaked around the Miocene–Pliocene transition (ca 5.3 mya), altering atmospheric circulation and enhancing the South Asian monsoon system (Morley 2018). These climatic shifts led to more pronounced wet and dry seasons, favouring the expansion of grasslands, savannas, and seasonal forests, while fragmenting evergreen forests. This fragmentation likely isolated populations of evergreen forest‐dependent species such as *Ratufa*. The niche modelling analysis supports this scenario, revealing two isolated areas of high suitability for *R. bicolor* in mainland SE Asia during the Pliocene. These areas spatially align with this species' “North” and “South” major lineages (Figures 1a and 3f), suggesting that these populations likely diverged in allopatry within isolated evergreen forest pockets during this period. Furthermore, we cannot rule out an interplay of factors causing the divergence of these two lineages, including both habitat fragmentation and river vicariance. The Mekong River began forming its modern drainage system during this time frame (ca 5–2 mya). The river likely connected its upper and lower segments, forming a continuous flow from the Tibetan Plateau to the South China Sea. The “north” and “south” major lineages of *R. bicolor* diverged around this time (5.25 mya) and the distribution limits of these two lineages also seem to be spatially congruent with most of the course of the Mekong River (Figure 1). The Mekong and other large rivers in SEA and southern China are thought to represent major vicariance agents, isolating populations of different small vertebrate taxa (He et al. 2019; Klabacka et al. 2020; Hinckley et al. 2023; Hinckley, Maldonado, et al. 2024). This speciation event is aligned temporally (and in some cases also spatially congruent) with divergence events in other Asian mammals, including the Sundaic and Indochinese clades within *Crocidura* and *Chimarrogale*, *Trachypithecus* species groups, *Ochotona* spp., *Dremomys rufigenis sensu lato* major clades, *Tamiops swinhoei‐T. maritimus*, and *Hylomys peguensis‐H. macarong* (Abramov et al. 2017; Abd Wahab et al. 2020; Roos et al. 2020; Ge et al. 2023; Hinckley et al. 2022, 2023; Hinckley, Camacho‐Sanchez, et al. 2024). The Indian subcontinent species *R. macroura* and *R. indica* also diverged during this time of C4 grassland domination, as highlighted by a replacement of mammalian frugivores and browsers to grazers (Patnaik 2015; Morley 2018). We hypothesize that the extinction of dipterocarps over most of their former range may have contributed to the divergence in allopatry in the two last dipterocarp refugia in the Indian subcontinent during this period: western Ghats ( *R. indica* ) and Sri Lanka ( *R. macroura* ; Gunatilleke et al. 2017). These ancient speciation events in *Ratufa* are synchronous with the two peaks of diversification (mid‐Miocene and Miocene–Pliocene boundary) described in the Tibeto‐Himalayan region (Mosbrugger et al. 2018).

During the Late Pliocene (ca. 3 mya), there was an almost simultaneous dispersal or vicariance event across Sundaland in both taxa (Figure 2b). In *R. bicolor* , a “mainland” lineage including southeastern mainland Asia, Anambas, and Natunas populations, diverged from Sumatra and Java plus Bali lineages. Short internal branches and low concordance factors indicate a hard polytomy and possible incomplete lineage sorting, suggesting a rapid diversification event (Figure S3). However, introgression between sister lineages cannot be ruled out based on the current evidence. In *R. affinis* , the east and west Sunda lineages diverged around the same time. It is thought that the exposure of the Sunda shelf fostered physical connectivity during this time (Morley 2018; Husson et al. 2020). Cooling paleoclimates have also been linked to a significant drop in global dipterocarp diversification rates and a potential crisis among evergreen forest‐adapted flying squirrels, according to several studies (Casanovas‐Vilar et al. 2018; Bansal et al. 2022). Therefore, it is plausible that lineages diverged within rainforest pockets across an exposed shelf acting as ecological barriers to gene flow, rather than in isolated landmasses separated by marine barriers (Heaney 1991; Bird et al. 2005; Sheldon et al. 2015). During the Early Pleistocene periods, when rainforest expanded and potential physical and habitat connectivity increased, the North Sunda Paleoriver (NSP) might have played a key role in maintaining east–west faunal differentiation (Voris 2000; Cheng and Faidi 2024). The NSP potentially serves as a barrier and distributional limit for both *R. affinis* and *R. bicolor* . It marks the furthest east location of *R. bicolor* (Pulau Lagong, just northwest of NSP) and the break between eastern and western lineages of *R. affinis* , which extend to the North and South Natunas, respectively. These findings mirror an emerging pattern recently showcased in birds but provide further geographic resolution due to the inclusion of populations flanking the NSP to the north and south (Garg et al. 2022; Berman et al. 2024). Other paleorivers, such as the paleo‐Mekong, delineate the northeast limit of *R. bicolor* “South” lineage (Con Dao Islands) and the southeastern limit of the “North” lineage (Figure 1a). The Straits of Malacca paleoriver represents the distribution boundary between the “mainland” and “Sumatra” *R. bicolor* lineages (Voris 2000; Cheng and Faidi 2024). It is suggested that grasslands associated with these rivers may have been primary open habitats during Pleistocene low stands, acting as both physical and ecological barriers to gene flow across diverging allopatric lineages (Morley 2018). The presence of riverine grasslands instead of seasonal forests/savannas, would have promoted the isolation and divergence in both the rainforest specialist ( *R. affinis* ) and also the more seasonal‐adapted, yet still forest‐dependent species complex ( *R. bicolor* ). Additionally, the dry climate of southern Sumatra, in combination with the NSP, might have served as a dispersal barrier between eastern and western *R. affinis* lineages (Figure 3c). This could explain why populations from Bangka/Belitung islands, despite their proximity to Sumatra, are frequently genetically clustered within the diversity of Bornean populations in *R. affinis* and other taxa (Mason et al. 2019; Dixit et al. 2023). In summary, this interplay of physical and ecological barriers associated with rivers and the drier regions of South Sumatra, likely played a central role in promoting speciation within *R. affinis* and *R. bicolor* in Sundaland.

Species‐level diversity within *R. bicolor* and *R. affinis* appears to be underestimated. Based on our results, these taxa should be treated as species complexes until species boundaries can be more thoroughly evaluated with additional lines of evidence, such as morphological and ecological data. Four clades within *R. bicolor* diverged between 5 and 3 mya, while two clades within *R. affinis* diverged around 3 mya. These divergence times are greater than or fall within the average speciation timeframe observed in many mammalian taxa (Hawkins et al. 2016; Hinckley et al. 2022; Gu et al. 2023; Orkin et al. 2024; Castañeda‐Rico et al. 2025), suggesting that the current diversity within *Ratufa* could potentially double from four to eight recognised species. In fact, the 5 my divergence between the northern and southern lineages of *R. bicolor* exceeds that observed between the currently recognised species *R. indica* and *R. macroura* . The distribution of these two *R. bicolor* clades overlaps in northwestern Thailand, suggesting potential reproductive isolation. Given the habitat connectivity during the Pleistocene and divergence times predating this period, it is likely that some degree of reproductive isolation had already been established. *Ratufa indica* and *R. macroura* are also partially sympatric in southern India, but in contrast with the two *R. bicolor* clades, these are thought to hybridise in a few places (Thomas et al. 2018; Sankari et al. 2023). The two major *R. bicolor* clades are externally diagnosable. Northern populations are characterised by the presence of ear tufts and the absence of flash marks on the forearm dorsum (Figure S8), whereas southern populations exhibit the opposite pattern (Figure S9). The oldest available name for the northern lineage (‘purple’ clade in Figures 1 and 2) is *Ratufa gigantea* (McClelland in Horsfield, 1840), currently treated as a subspecies of *R. bicolor* (Ellerman and Morrison‐Scott 1951; Moore and Tate 1965; Corbet and Hill 1992; Thorington Jr et al. 2012; Koprowski et al. 2016), but previously recognised as a distinct species (Bonhote 1900; Thomas 1908; Robinson and Kloss 1919; Pocock 1923; Allen 1925; Delacour 1940). Based on our new findings, we here revalidate *R. gigantea* as a distinct species. Additional molecular species delimitation analyses and the integration of other lines of evidence will be pivotal in evaluating the specific status of the more recently diverged allopatric lineages (ca. 3 mya). We currently consider the southern mainland Asia and Sumatra lineages within *R. bicolor sensu stricto–‘green’* and “yellow” clades in Figures 1 and 2– as candidate species, along with the eastern lineage of *R. affinis–the ‘orange’* clade in Figures 1 and 2.

A separate aspect of the results from this study highlights the challenges inherent in the field of museum genomics (Raxworthy and Smith 2021). We detected a potentially important negative synergistic effect of historic DNA (hDNA) and UCE structure (Hosner et al. 2016). The fragmented nature of hDNA led to shorter sequences and the loss of informative sites present in flanking regions of UCEs. For many loci, only the uninformative core region is retained in degraded samples. This results in a high number of poorly resolved gene trees, low gCF and high gDFP. The prevalence of short average contigs has been shown to bias gene tree inference and cause erroneous results in gene tree reconciliation in other studies (Hosner et al. 2016). In this context, we caution researchers to consider this methodological bias to avoid misleading interpretations. We emphasise the importance of evaluating the robustness of genetic patterns in hDNA by sequencing multiple samples from each operational unit, scanning for contamination, filtering datasets to make them more comparable, and evaluating the effect of missing data (Raxworthy and Smith 2021; Abreu et al. 2022). For divergence dating studies with hDNA, we recommend additional filtering to minimise missing data, followed by SPRUCEUP (Borowiec 2019) and manual inspection and editing of the input alignment to overcome divergence overestimation through the removal of short outlier sequences (1–3 bp) (see methods), as performed in this study.

Beyond the technical limitations of hDNA, our results suggest dwarfism evolved independently and rapidly across three separately evolving lineages within *Ratufa* (Figure 1 and Figure S7). More specifically, it evolved in *R. bicolor* from Con Dao (Con Son Island), “west” *R. affinis* from North Natunas (Pulau Laut & Pulau Natuna Besar), and “east” *R. affinis* from South Natunas (Pulau Serasan). This convergence in body size might have been an adaptation to limited resources within small island areas (Heaney 1978). All of these represent relatively remote and small islands—two major predictors of insular dwarfism in mammals (Heaney 1978; Lomolino 2005; Sargis et al. 2018; Benítez‐López et al. 2021). On such islands, reduced forest extent and resource availability constrain carrying capacity (Sargis et al. 2018). Larger individuals, which require extensive home ranges and higher energetic intake, may therefore be at a selective disadvantage. In contrast, resource competition (both intra‐ and interspecific) can favor smaller individuals that are able to subsist on limited resources and exploit smaller habitat patches more efficiently. Moreover, insular environments often experience relaxed predation pressure, a condition that can further promote shifts in life‐history strategies and body size evolution (Heaney 1978; Lomolino 2005). Interestingly, it was revealed that colugos also experienced at least five cases of island dwarfism in different Sundaic satellite islands (Adang, Langkawi, Aur, Bakong, and Karimata; Mason et al. 2019) and many other examples of rapid dwarfism evolution have been revealed in other regions (Lister 1989; Anderson and Handley Jr 2002; Rozzi and Lomolino 2017).

## Author Contributions

A.H.: conceptualization, funding acquisition, data generation/curation, formal analysis (phylogenomics), visualisation, writing (original draft, review and editing); G.E.P.‐B.: formal analysis (ecological niche modelling), visualisation, writing (original draft: ecological niche modelling methods and results, review and editing); J.E.M.: conceptualization, funding acquisition, writing (review and editing); M.F.C.F.: data generation/curation, writing (review and editing); J.A.E.: funding acquisition, writing (review and editing); N.I.: funding acquisition, writing (review and editing); M.T.R.H.: conceptualization, funding acquisition, visualisation, writing (review and editing). All authors gave final approval for publication and agreed to be held accountable for the work performed therein.

## Disclosure

*Benefit Sharing‐Statement*: The Nagoya Protocol does not apply to this research, as all genetic samples were obtained from museum specimens housed in institutions within the United States and predate the initiation of Nagoya. Nonetheless, we have prioritized equitable research practices: a scientist from one of the source countries from where the museum specimens originated is an active collaborator and coauthor on this study. Research findings have been shared with relevant provider communities and the broader scientific community. The study directly addresses a priority concern—the conservation of the focal taxa. More broadly, our research group is committed to fostering international scientific partnerships and institutional capacity building. As part of this commitment, a Smithsonian NSF‐funded museum genomics workshop was recently held to support skills exchange and collaboration.

## Ethics Statement

This work did not require ethical approval from a human subject or animal welfare committee.

## Conflicts of Interest

The authors declare no conflicts of interest.

## Supporting information


**Appendix S1:** mec70179‐sup‐0001‐appendixS1.docx.

**Table S1:** Specimens included in the phylogenomic analyses and associated metadata.

**Table S2:** Summary characteristics of the ultraconserved element (UCE) datasets analysed in this study.

**Table S3:** Specimen and citizen‐science occurrence records used for the species distribution modelling analyses.

**Table S4:** Occipitonasal skull length measurements and associated specimen information.

**Table S5:** Permutation importance of bioclimatic variables for *Ratufa affinis* and *R. bicolor* .

## Data Availability

Genetic data: All datasets and configuration files generated in this study are currently available in the Supporting Information or in the following repository: Figshare. These genetic sequences could not be submitted to NCBI in time due to the U. S. government shutdown and lack of response from the European Nucleotide Archive. However, they are available on Figshare and will be uploaded to the appropriate repositories as soon as possible. Sample metadata: Related metadata can be found in Table S1 (including georeferences in decimal degrees and date of collection) and museum catalogue numbers can be matched to both the deposited genetic data and deposited metadata. Niche modelling data: All distribution records and associated metadata can be found in Table S3. Morphological data: All morphometric data can be found in Table S4.
