# Supplementary material for: Pliocene Forest Fragmentation Shaped Speciation in Tropical Asia's Giant Squirrels (Ratufa)
Source: Mol Ecol. 2025 Nov 25;34(24):e70179. doi: 10.1111/mec.70179 (PMC12717977; doi:10.1111/mec.70179)
Supplement: Supplementary file 1 — Appendix S1: mec70179‐sup‐0001‐appendixS1.docx. [file MEC-34-e70179-s001.docx]

**Supplemental Information for:**

**Pliocene forest fragmentation shaped speciation in Tropical Asia’s giant squirrels (*Ratufa*)**

Arlo Hinckley, Gonzalo E. Pinilla-Buitrago, Jesús E. Maldonado, Mary Faith C. Flores, Jacob Esselstyn, Nurul Inayah, Melissa T. R. Hawkins

**Table of Contents:**

| **Input partition for mitogenome ML analyses** | Pages 2-3 |
| --- | --- |
| **Best partition scheme and model selection**  **Information for mitogenome ML analyses;**  **Likelihood and BIC scores** | Pages 3-4 |
| **Bayes factor analyses for different BEAST runs** | Pages 5-6 |
| **Figure S1. SVDquartets UCE phylogeny** | Page 7 |
| **Figure S2. ASTRAL UCE phylogeny** | Page 8 |
| **Figures S3-S5. Gene and site concordance analyses** | Pages 9-11 |
| **Figure S6. Niche overlap between *R. affinis* and**  ***R. bicolor* species complexes** | Page 12 |
| **Figures S7-S9. Morphological variation in *R. affinis* and**  ***R. bicolor* species complexes** | Pages 13-15 |

**Input partition for mitogenome ML analyses**

#nexus

begin sets;

charset tRNA_Phe=1-71;

charset 12S=72-1046;

charset tRNA_Val= 1047-1119;

charset 16S=1120-2729;

charset tRNA_Leu=2730-2808;

charset ND1_1stpos=2809-3763\3;

charset ND1_2ndpos=2810-3763\3;

charset ND1_3rdpos=2811-3763\3;

charset tRNA_Ile_gln_met=3764-3975;

charset ND2_1stpos=3976-5017\3;

charset ND2_2ndpos=3977-5017\3;

charset ND2_3rdpos=3978-5017\3;

charset tRNA_trp_tyr= 5018-5413;

charset COX1_1stpos=5414-6958\3;

charset COX1_2ndpos=5415-6958\3;

charset COX1_3rdpos=5416-6958\3;

charset tRNA_ser_asp =6959-7104;

charset COX2_1stpos=7105-7788\3;

charset COX2_2ndpos=7106-7788\3;

charset COX2_3rdpos=7107-7788\3;

charset tRNA_lys=7789-7862;

charset ATP8_1stpos=7863-8023\3;

charset ATP8_2ndpos=7864-8023\3;

charset ATP8_3rdpos=7865-8023\3;

charset ATP6_1stpos=8024-8703\3;

charset ATP6_2ndpos=8025-8703\3;

charset ATP6_3rdpos=8026-8703\3;

charset COX3_1stpos=8704-9487\3;

charset COX3_2ndpos=8705-9487\3;

charset COX3_3rdpos=8706-9487\3;

charset tRNA_Gly=9488-9559;

charset ND3_1stpos=9460-9906\3;

charset ND3_2ndpos=9461-9906\3;

charset ND3_3rdpos=9462-9906\3;

charset tRNA_Arg=9907-9976;

charset ND4L_1stpos=9977-10266\3;

charset ND4L_2ndpos=9978-10266\3;

charset ND4L_3rdpos=9979-10266\3;

charset ND4_1stpos=10267-11644\3;

charset ND4_2ndpos=10268-11644\3;

charset ND4_3rdpos=10269-11644\3;

charset tRNA_lys_leu=11645-11845;

charset ND5_1stpos=11846-13647\3;

charset ND5_2ndpos=11846-13647\3;

charset ND5_3rdpos=11846-13647\3;

charset tRNA_glu=13648-13720;

charset CYTB_1stpos=13721-14860\3;

charset CYTB_2ndpos=13722-14860\3;

charset CYTB_3rdpos=13723-14860\3;

charset tRNA_pro=14861-15005;

charset ND6_1stpos=15006-15533\3;

charset ND6_2ndpos=15007-15533\3;

charset ND6_3rdpos=15008-15533\3;

end;

**Best partition scheme and model selection information for mitogenome ML analyses**

#nexus

begin sets;

  charset tRNA_Phe_12S_tRNA_Val_16S_ND2_1stpos_tRNA_ser_asp_ATP8_1stpos_ATP8_2ndpos_ATP6_1stpos_ND3_2ndpos_ND4L_1stpos_ND4_1stpos_tRNA_lys_leu_ND5_1stpos_ND5_2ndpos_ND5_3rdpos_tRNA_glu = 1-71  72-1046  1047-1119  1120-2729  3976-5017\3  6959-7104  7863-8023\3  7864-8023\3  8024-8703\3  9461-9906\3  9977-10266\3  10267-11644\3  11645-11845  11846-13647\3  11846-13647\3  11846-13647\3  13648-13720;

  charset tRNA_Leu_ND1_1stpos_tRNA_Ile_gln_met_tRNA_trp_tyr_COX1_1stpos_COX2_1stpos_tRNA_lys_COX3_1stpos_CYTB_1stpos = 2730-2808  2809-3763\3  3764-3975  5018-5413  5414-6958\3  7105-7788\3  7789-7862  8704-9487\3  13721-14860\3;

  charset ND1_2ndpos_COX1_2ndpos_COX2_2ndpos_ATP6_2ndpos_COX3_2ndpos_ND4L_2ndpos_ND4_2ndpos_CYTB_2ndpos_ND6_2ndpos = 2810-3763\3  5415-6958\3  7106-7788\3  8025-8703\3  8705-9487\3  9978-10266\3  10268-11644\3  13722-14860\3  15007-15533\3;

  charset ND1_3rdpos_ND2_3rdpos_ND4L_3rdpos_ND4_3rdpos = 2811-3763\3  3978-5017\3  9979-10266\3  10269-11644\3;

  charset ND2_2ndpos_ND3_3rdpos = 3977-5017\3  9462-9906\3;

  charset COX1_3rdpos_COX2_3rdpos_ATP8_3rdpos_ATP6_3rdpos_COX3_3rdpos_ND3_1stpos = 5416-6958\3  7107-7788\3  7865-8023\3  8026-8703\3  8706-9487\3  9460-9906\3;

  charset tRNA_Gly_tRNA_Arg_tRNA_pro = 9488-9559  9907-9976  14861-15005;

  charset CYTB_3rdpos = 13723-14860\3;

  charset ND6_1stpos = 15006-15533\3;

  charset ND6_3rdpos = 15008-15533\3;

  charpartition mymodels =

    TIM2+F+I+R3: tRNA_Phe_12S_tRNA_Val_16S_ND2_1stpos_tRNA_ser_asp_ATP8_1stpos_ATP8_2ndpos_ATP6_1stpos_ND3_2ndpos_ND4L_1stpos_ND4_1stpos_tRNA_lys_leu_ND5_1stpos_ND5_2ndpos_ND5_3rdpos_tRNA_glu,

    TIM2+F+I+G4: tRNA_Leu_ND1_1stpos_tRNA_Ile_gln_met_tRNA_trp_tyr_COX1_1stpos_COX2_1stpos_tRNA_lys_COX3_1stpos_CYTB_1stpos,

    TN+F+R2: ND1_2ndpos_COX1_2ndpos_COX2_2ndpos_ATP6_2ndpos_COX3_2ndpos_ND4L_2ndpos_ND4_2ndpos_CYTB_2ndpos_ND6_2ndpos,

    TN+F+I+G4: ND1_3rdpos_ND2_3rdpos_ND4L_3rdpos_ND4_3rdpos,

    HKY+F+I+G4: ND2_2ndpos_ND3_3rdpos,

    TN+F+I+G4: COX1_3rdpos_COX2_3rdpos_ATP8_3rdpos_ATP6_3rdpos_COX3_3rdpos_ND3_1stpos,

    HKY+F+G4: tRNA_Gly_tRNA_Arg_tRNA_pro,

    GTR+F+I+G4: CYTB_3rdpos,

    GTR+F+I+G4: ND6_1stpos,

    GTR+F+I+G4: ND6_3rdpos;

end;

**Likelihood and BIC scores of IQTREE analyses**

Edge-proportional partition model (-spp)

Log-likelihood of the tree: -77802.3692 (s.e. 690.4481)

Bayesian information criterion (BIC) score: 157990.0675

Edge-unlinked partition model (-sp)

Log-likelihood of the tree: -79106.5994 (s.e. 694.7017)

Bayesian information criterion (BIC) score: 161477.3335

**Bayes factor analyses for different BEAST runs**

HKY+G4 triple calibration strict clock

Step        theta         likelihood   contribution ESS

0            1            -244831.1218 0            5.9054

1            0.5982       -244830.8981 -98372.9698  88.4304

2            0.3258       -244835.6091 -66699.6702  17.1035

3            0.1548       -244845.4273 -41850.5562  38.3361

4            0.0593       -244856.9419 -23380.3407  63.9315

5            0.0154       -244898.2701 -10771.8702  44.7487

6            0.0015       -244936.9981 -3389.2224   52.3089

7            0            -245074.6051 -373.5044    36.589

sum(ESS) = 347.3535

marginal L estimate = -244838.13390656761

HKY+G4 triple calibration optimised relaxed clock

Step        theta         likelihood   contribution ESS

0            1            -244744.1295 0            29.2624

1            0.5982       -244744.5532 -98338.4542  23.255

2            0.3258       -244746.2159 -66675.157   6.6788

3            0.1548       -244751.6077 -41835.0932  11.3215

4            0.0593       -244751.8494 -23370.6804  11.7578

5            0.0154       -244765.1123 -10766.3805  8.1678

6            0.0015       -244764.9078 -3386.9615   6.3261

7            0            -244779.2238 -373.0619    8.0899

sum(ESS) = 104.8594

marginal L estimate = -244745.7887773491

# Define the marginal likelihoods

marginal_L_strict_clock = -244838.13

marginal_L_relaxed_clock = -244745.79

# Calculate the Bayes Factor (BF)

bayes_factor = 2 * (marginal_L_relaxed_clock - marginal_L_strict_clock)

bayes_factor

The Bayes Factor (BF) comparing the relaxed clock model to the strict clock model is approximately 184.68. This suggests very strong support for the relaxed clock model over the strict clock model, as a Bayes Factor greater than 10 is generally considered strong evidence.

HKY+G4 Double calibration optimised relaxed clock

marginalLs[7 ] = -244771.04618560855

Step        theta         likelihood   contribution ESS

0            1            -244745.3341 0            11.9555

1            0.5982       -244746.3816 -98339.1006  36.1582

2            0.3258       -244748.7388 -66676.1454  13.0219

3            0.1548       -244750.5039 -41834.7972  15.3243

4            0.0593       -244758.4081 -23371.2329  8.2399

5            0.0154       -244759.1672 -10766.164   35.0195

6            0.0015       -244765.9587 -3386.9745   7.4589

7            0            -244771.0462 -373.0495    13.406

sum(ESS) = 140.5842

marginal L estimate = -244747.46401305337

The Bayes factor comparing the double calibration model and the triple calibration model is approximately 3.34. This indicates mild support in favor of the triple calibration model over the double calibration model.

bmodeltest triple calibration optimised relaxed clock

Step        theta         likelihood   contribution ESS

0            1            -244447.7597 0            19.4184

1            0.5982       -244452.0511 -98220.7599  30.4898

2            0.3258       -244451.5364 -66595.3529  33.9998

3            0.1548       -244458.5879 -41784.8287  13.2686

4            0.0593       -244461.7514 -23342.9295  25.5647

5            0.0154       -244465.8959 -10753.249   30.2942

6            0.0015       -244468.5119 -3382.8626   54.4889

7            0            -244481.7205 -372.6085    27.6854

sum(ESS) = 235.2098

marginal L estimate = -244452.59106855895

The Bayes Factor comparing the BModelTest triple calibration model to the HKY triple calibration model is approximately 586.4. This indicates very strong support for the BModelTest model over the HKY model.


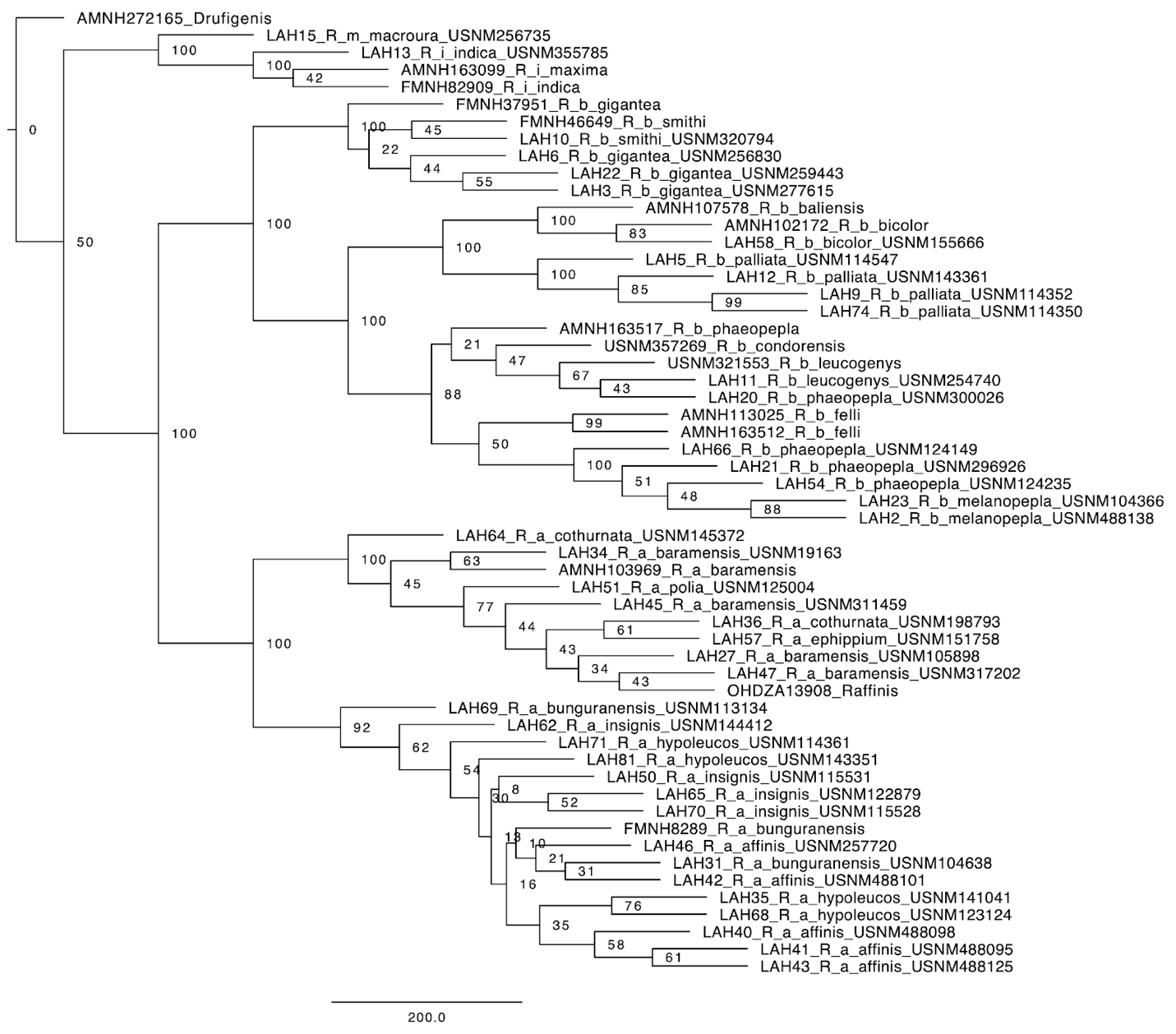


**Figure S1**. Phylogeny estimated with SVDquartets using a 75% complete matrix of 3224 ultraconserved elements loci. Nodal support is provided in bootstrap, which has high precision above a threshold of 70.


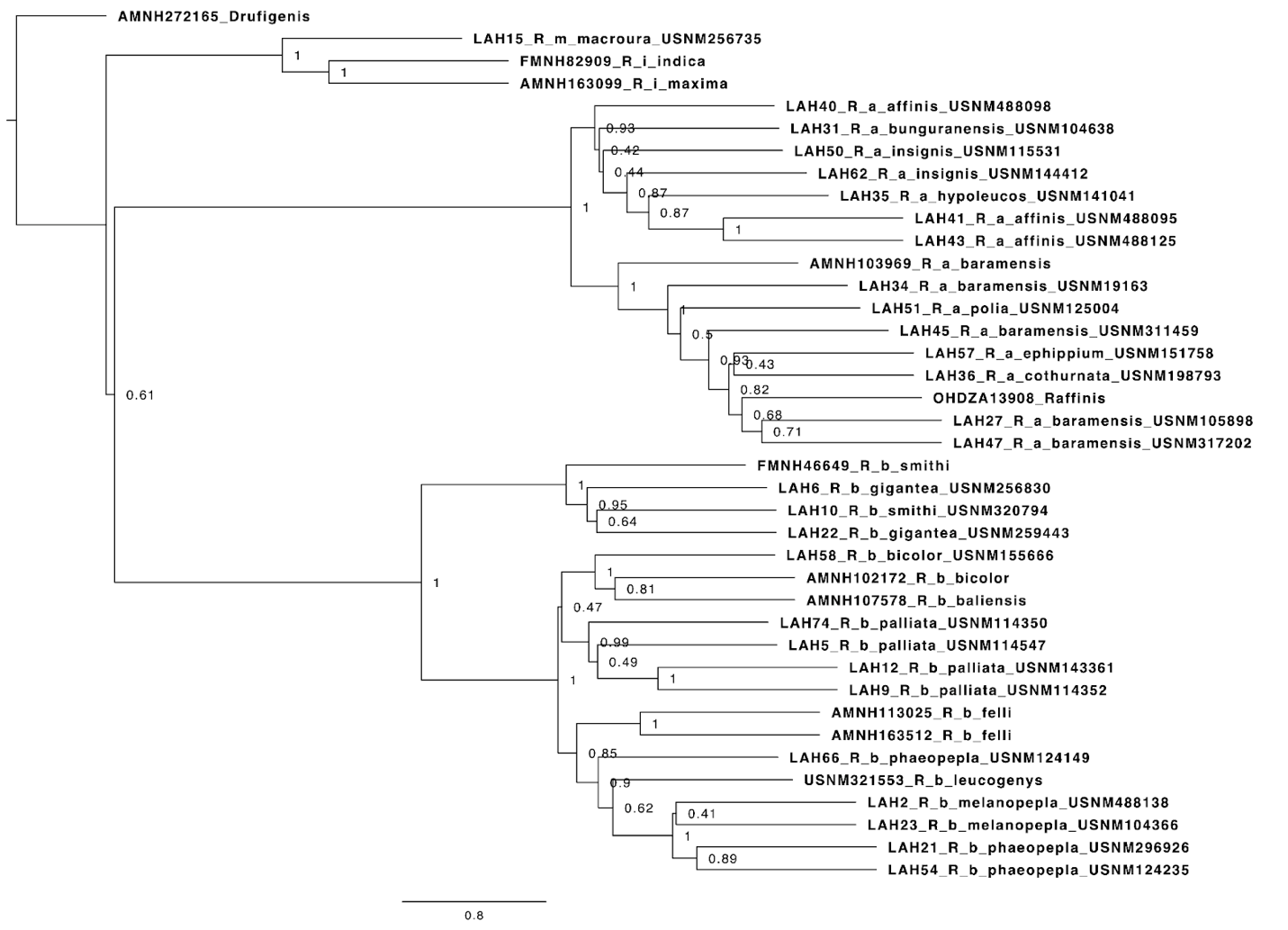


**Figure S2**. Phylogeny estimated with ASTRAL-III using 3224 individual UCE loci as gene trees. Node values represent the local posterior probability of the quadripartition, which has high precision above a threshold of 0.7 (Sayyari and Mirarab 2016).


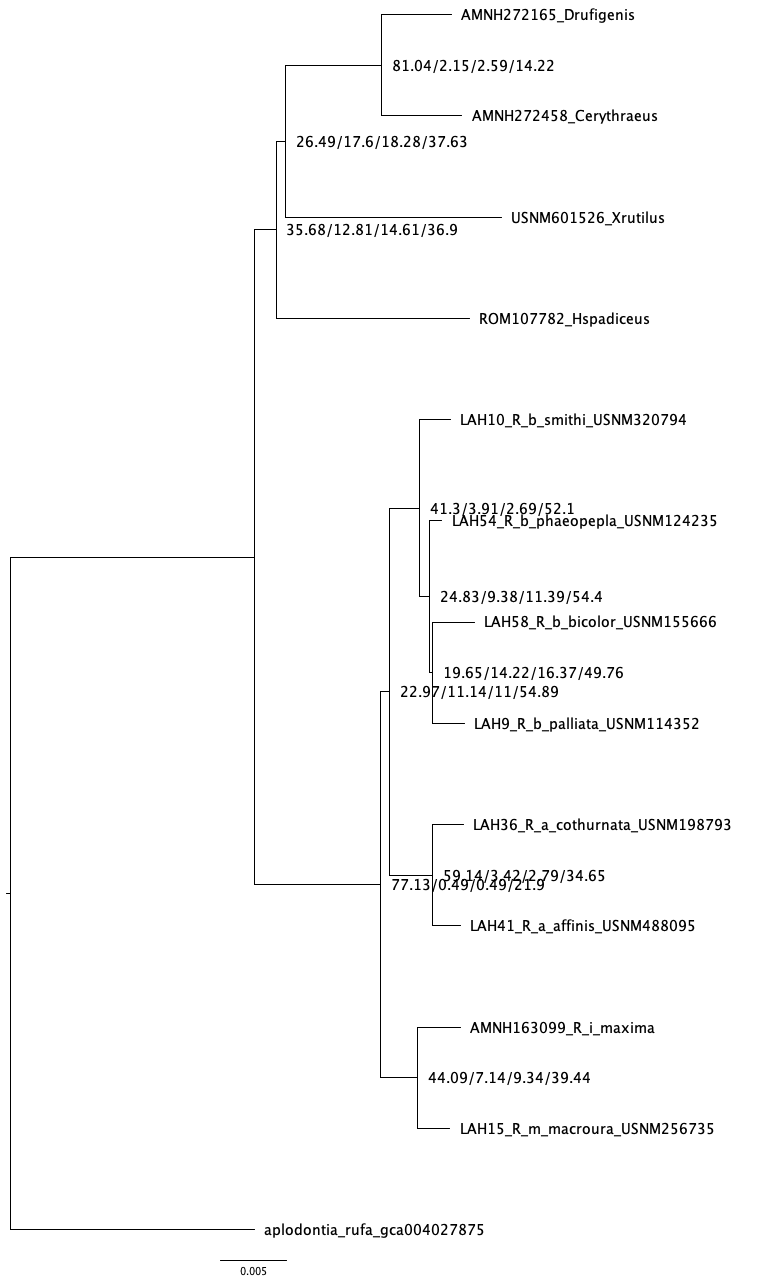


**Figure S3**. Maximum likelihood phylogenetic tree inferred with IQ-TREE. Node labels indicate gene concordance factors (gCF/gDF1/gDF2/gDFP). gCF represents the percentage of gene trees that support a specific branch (split) in the species tree. gDF1 indicates the percentage of gene trees that support the first most common discordant topology (alternative resolution) to the species tree at a particular branch. gDF2 represents the percentage of gene trees that support the second most common discordant topology for the branch. gDFP represents the percentage of gene trees that do not resolve the branch as a bifurcation but instead show a polytomy (unresolved or multifurcating node).


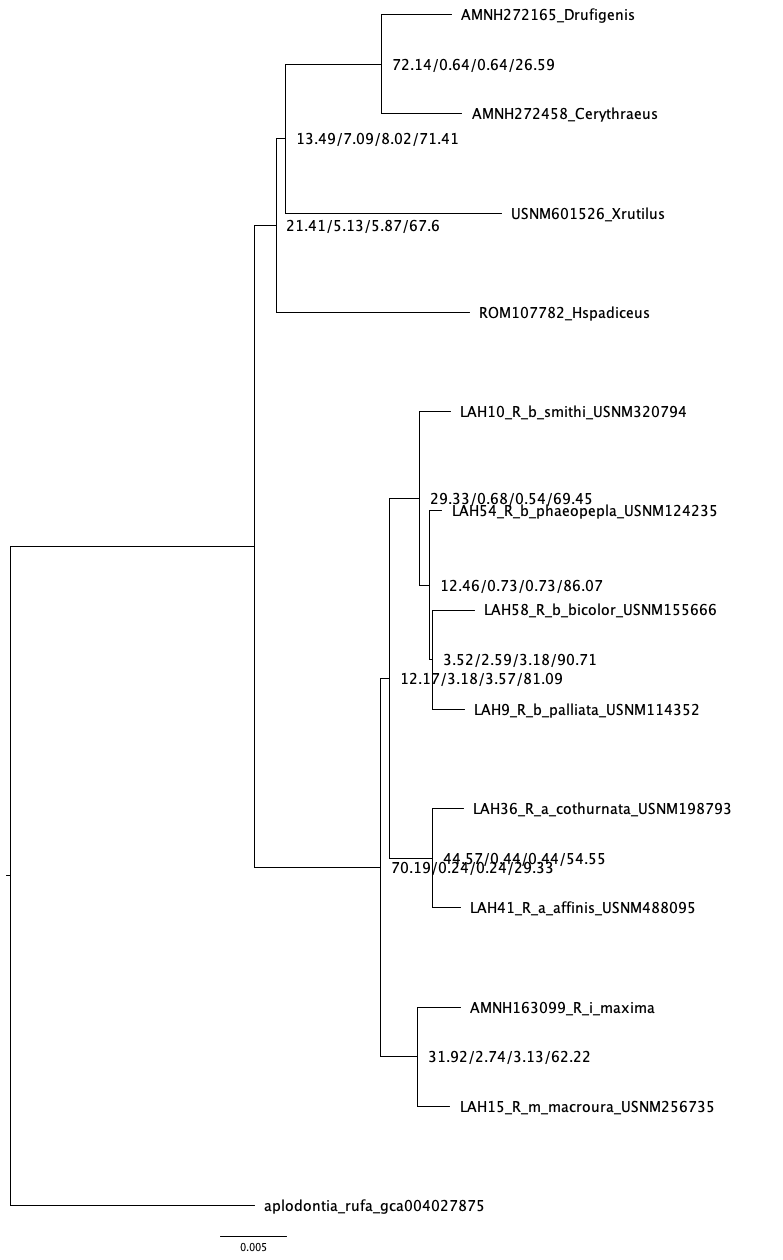


**Figure S4**. Maximum likelihood phylogenetic tree inferred with IQ-TREE. Low supported branches (UltraBP < 70) of input gene trees were contracted. Node labels indicate gene concordance factors (gCF/gDF1/gDF2/gDFP). gCF represents the percentage of gene trees that support a specific branch (split) in the species tree. gDF1 indicates the percentage of gene trees that support the first most common discordant topology (alternative resolution) to the species tree at a particular branch. gDF2 represents the percentage of gene trees that support the second most common discordant topology for the branch. gDFP represents the percentage of gene trees that do not resolve the branch as a bifurcation but instead show a polytomy (unresolved or multifurcating node).


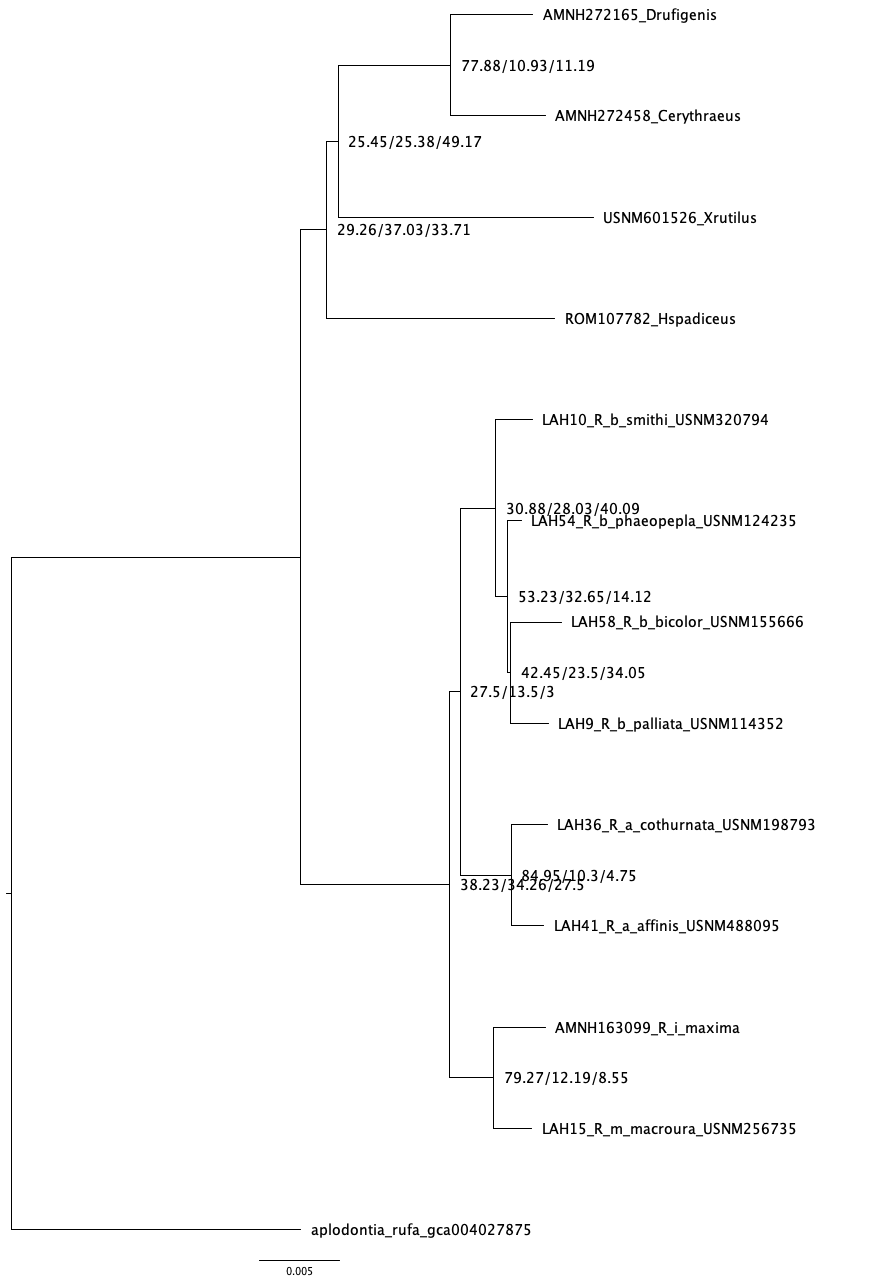


**Figure S5**. Maximum likelihood phylogenetic tree inferred with IQ-TREE. Node labels indicate site concordance factors (sCF/sDF1/sDF2). sCF is the percentage of sites supporting the clade defined by the branch. sDF1 and sDF2 represent the proportion of sites that support the "next-best" and “second-best” alternative topologies (the second/third most supported bipartition), respectively.


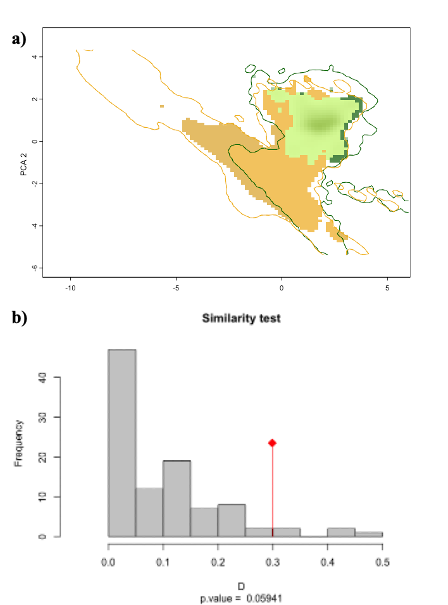


**Figure S6**. Niche overlap between *Ratufa* species complexes. The top panel illustrates niche overlap in environmental space, defined by the first two principal components (PCs) derived from occurrence and background points densities. Dark green represents the niche of *R. affinis*, while yellow represents the niche of *R. bicolor*. Areas highlighted in light green indicate regions of niche overlap between these taxa. The bottom histogram shows the null distribution of randomized Schoener's D values (or simply 'D values' if D is already defined), with the observed D value for the actual overlap highlighted in red.

**
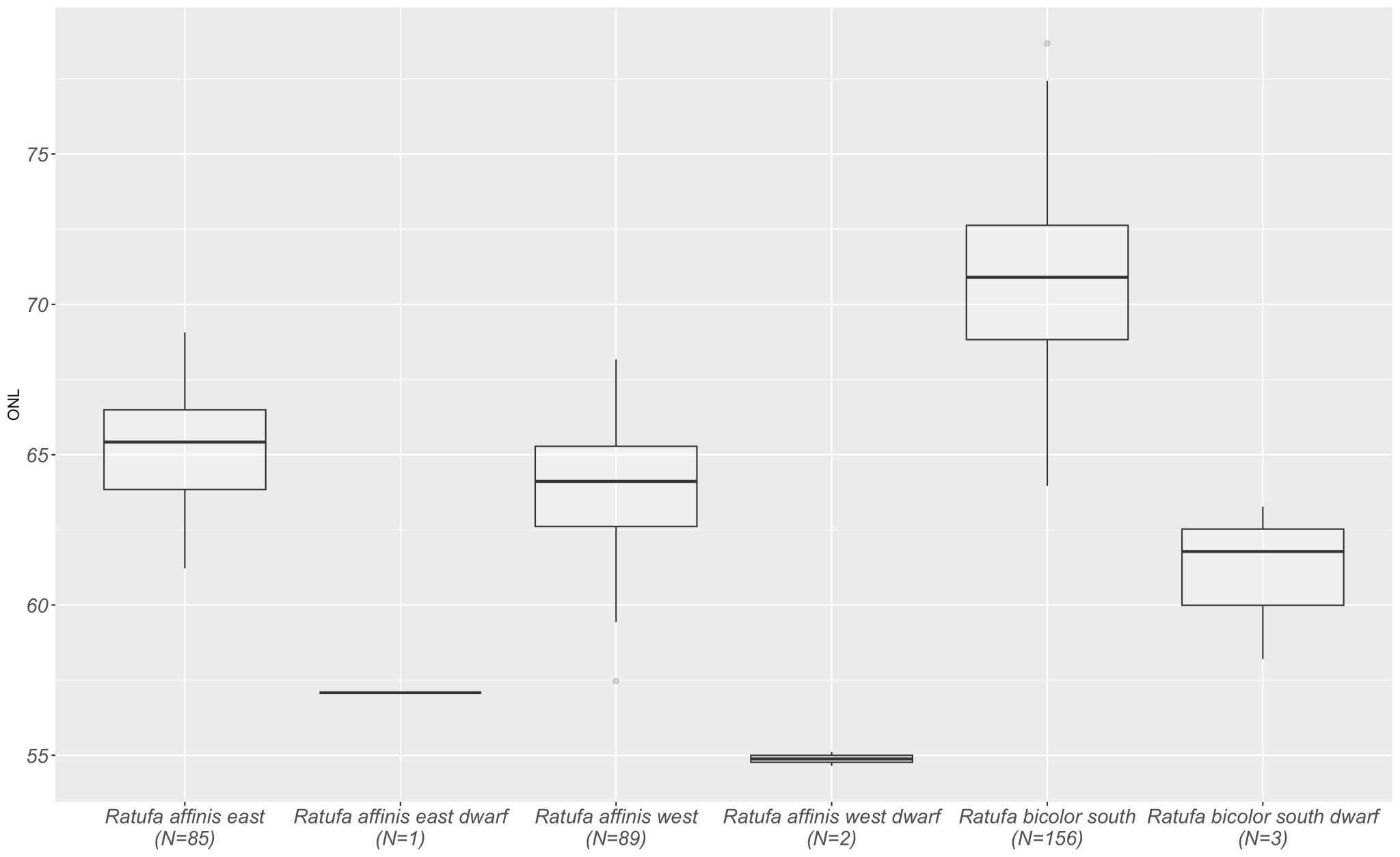
**

**Figure S7.** Differentiation in occipitonasal length (mm) between dwarf populations and their standard-sized relatives in two lineages of *R. affinis* and one of *R. bicolor.*


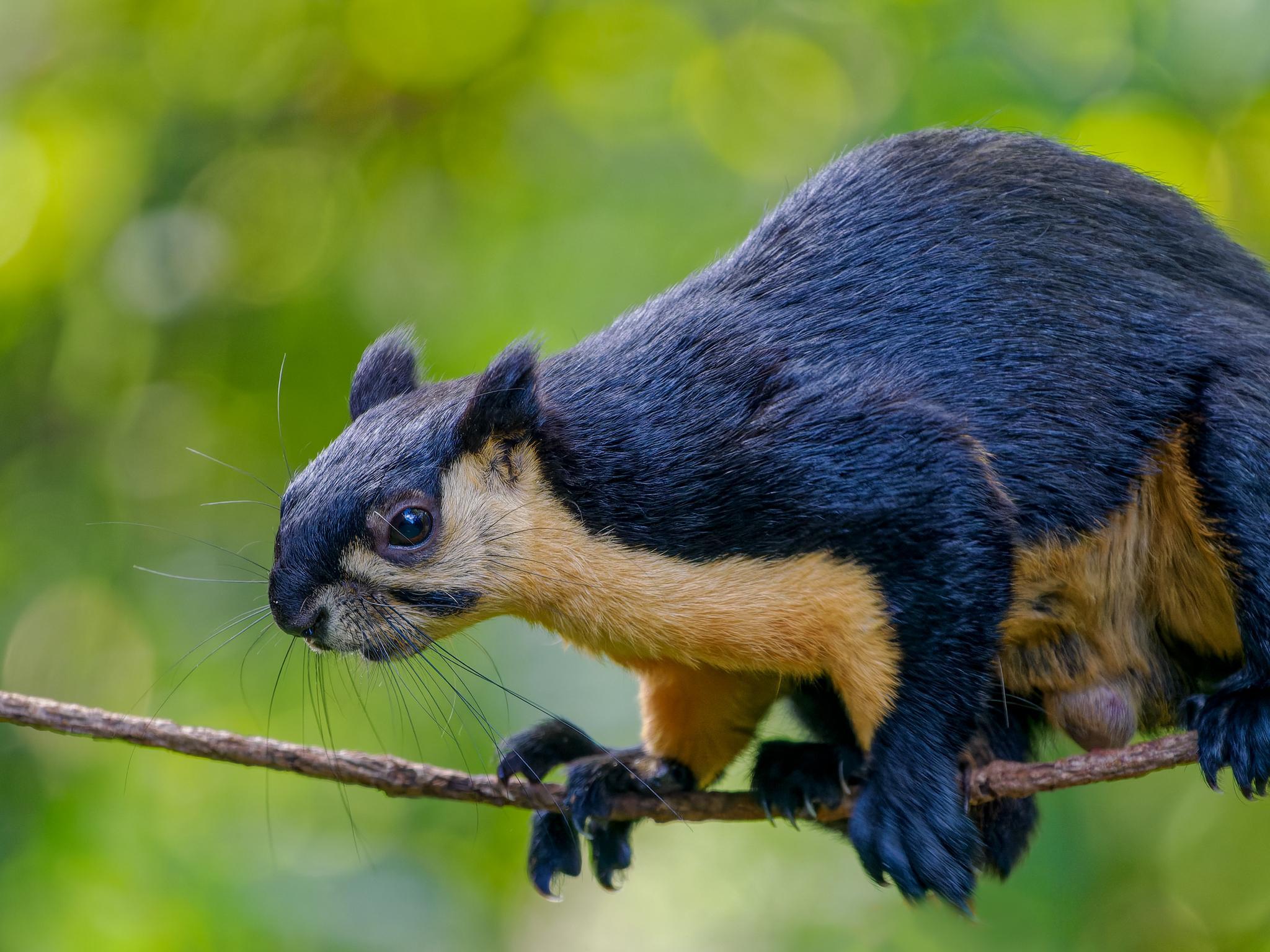


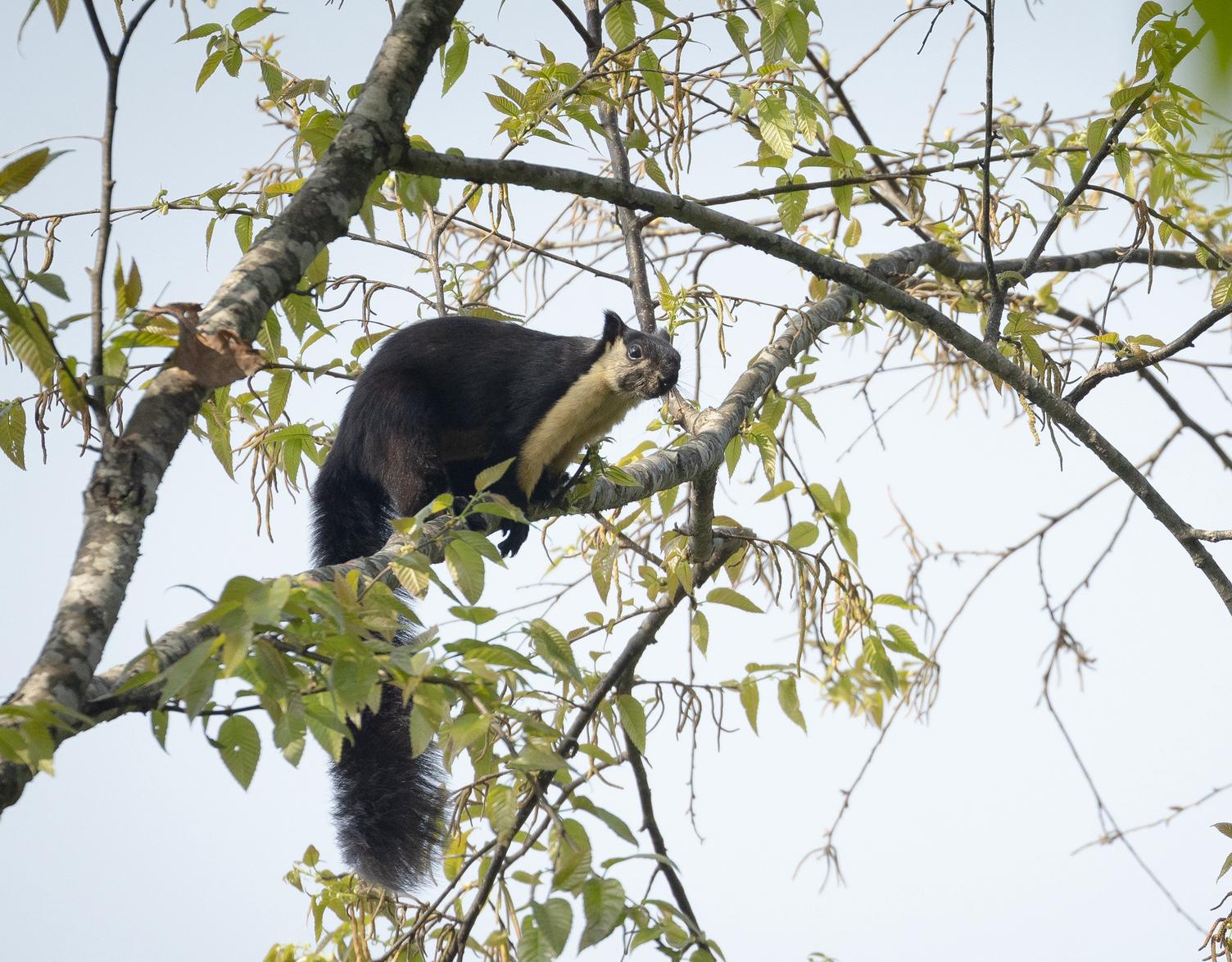


**Figure S8**. Live *Ratufa gigantea* photographed by Chen Gim Choon in Baoshan, China (top), and by Vincent Zhu in Myitkyina, Myanmar, <https://www.inaturalist.org/observations/255031612> (bottom).


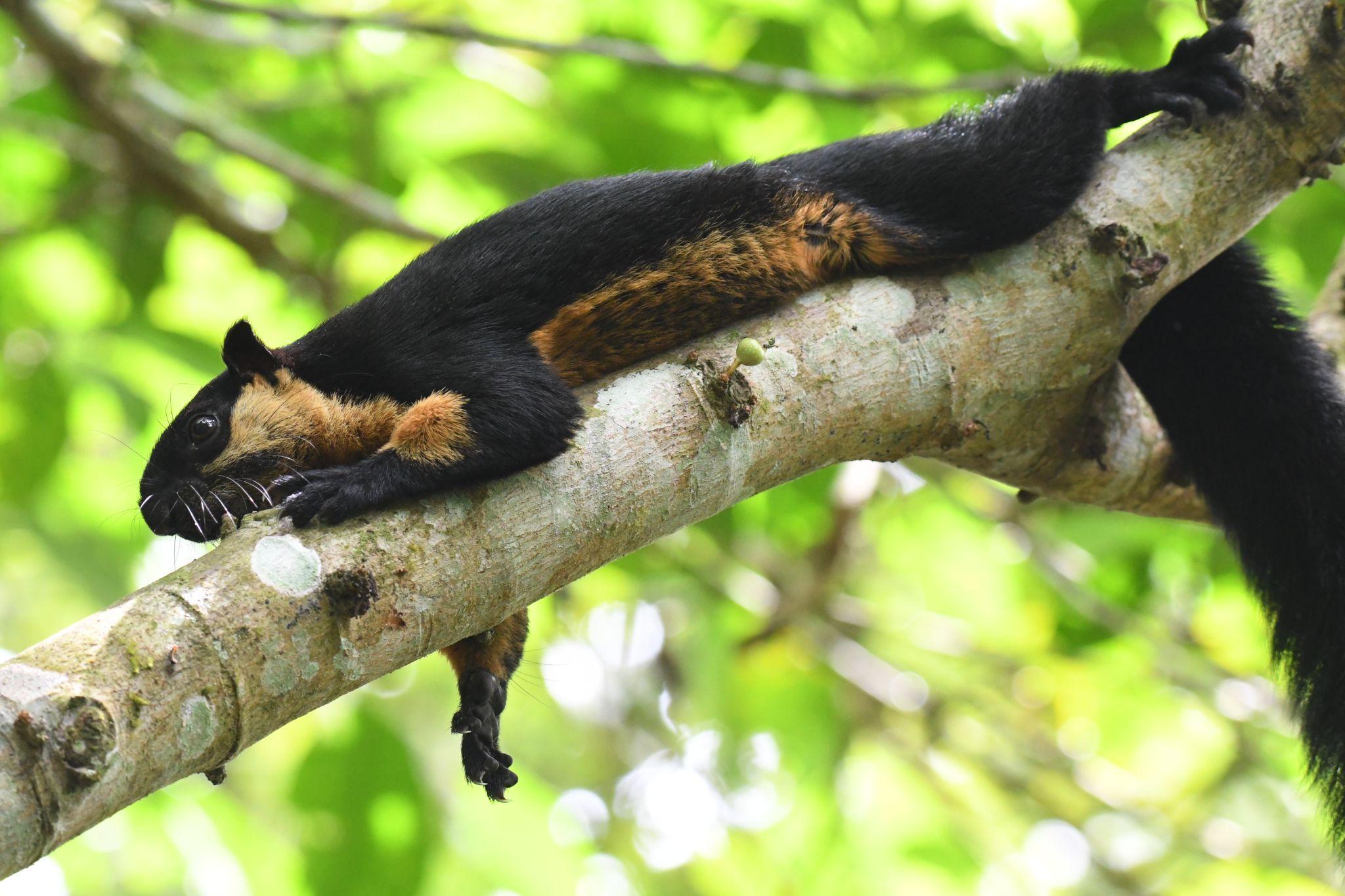


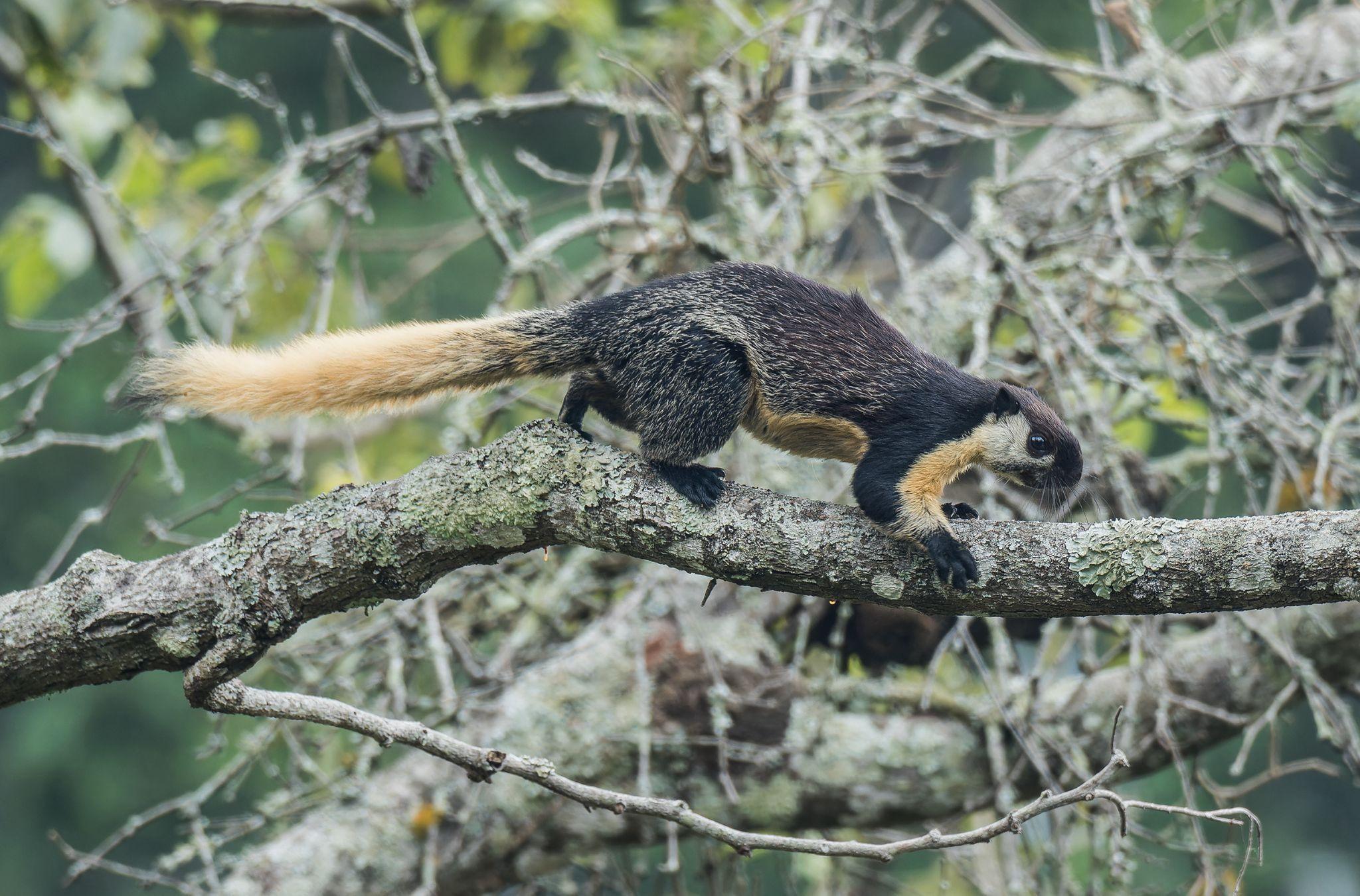


**Figure S9.** Live *Ratufa bicolor* photographed by Zachary Romano in Penang, Malaysia (top), and by Wich’yanan Limparungpatthanakij in Mount Gede, Java (bottom).
